# Nano MgO loaded thermosensitive HPCH@HA hydrogel accelerates in situ bone repair through osteoimmunomodulation while enhancing angiogenesis and osteogenesis

**DOI:** 10.1016/j.bioactmat.2025.08.007

**Published:** 2025-08-20

**Authors:** Feifei Ni, Longkang Chang, Yizhong Peng, Dongxu Li, Dong Wu, Kanglu Li, Xin Zhang, Xulin Jiang, Zengwu Shao, Yangyang Chen, Hong Wang

**Affiliations:** a Department of Orthopedics, The First Affiliated Hospital of Anhui Medical University, Hefei, 230022, China; b Department of Orthopedic, The First Affiliated Hospital of Zhengzhou University, Zhengzhou, 450052, China; c Department of Orthopedics, Union Hospital, Tongji Medical College, Huazhong University of Science and Technology, Wuhan, 430022, China; d Key Laboratory of Biomedical Polymers of Ministry of Education and Department of Chemistry, Wuhan University, Wuhan, 430072, China; e Department of Orthopaedics, The First Affiliated Hospital of Chongqing Medical University, Chongqing, 400016, China

**Keywords:** HPCH, Hydrogel, Bone repair, Osteoimmunomodulatory, Mg^2+^, Macrophage, T lymphocyte

## Abstract

Bone defect repair is a complex physiological process, starting with early modulation by the inflammatory immune system, and involves multiple physiological events, including angiogenesis, osteogenic differentiation, and mineralization. Biomaterial can regulate inflammatory responses through relevant immune cells in the local immune microenvironment of the implant-bone interface which is a hot topic in the field of regenerative medicine. Currently, Mg^2+^ regulates immune cells in the bone microenvironment to promote osteogenesis and angiogenesis mainly focuses on macrophages,but there is relatively little research on T cells.At the same time, the effective delivery and release of Mg^2+^ remains a challenge. To address these issues, we designed a new thermosensitive hyaluronic acid-hydroxypropyl chitin hydrogel (HPCH@HA) that has good affinity for Mg^2+^ and can sustained release it. In vitro, nano MgO loaded complex hydrogels effectively induced macrophage polarization from M0 phenotype to M2 phenotype and simultaneously activate T lymphocytes which also promoted human adipose-derived stem cells (hADSCs) osteogenic differentiation and mineralization and human umbilical vein endothelial cells (HUVECs) angiogenesis. In vivo, at the early stage of repair, the composite hydrogel has a good repair effect on mouse skull critical defect. All these results show that our designed composite hydrogels can effectively regulate the immune microenvironment of bone tissue and promoting the formation of mature bone in large bone defects and supporting in situ bone regeneration without the use of exogenous cells or inducers. It's a promising candidate as immunomodulatory biomaterials for bone tissue engineering purposes.

## Highlights

• Developed a nano MgO loaded thermosensitive hyaluronic acid-hydroxypropyl chitin hydrogel (HPCH@HA) for bone repair.
• Effective nano MgO Delivery: The HPCH@HA hydrogel exhibits strong affinity and provides sustained release of Mg^2+^ ions.
• Nano MgO composite hydrogel induces polarization of macrophages towards anti-inflammatory M2 phenotype.
• Nano MgO composite hydrogel nhances anti-inflammatory cytokine production and T lymphocyte activation.
• Nano MgO composite hydrogel promotes osteogenic differentiation and mineralization of hADSCs, and stimulates angiogenesis in HUVECs.

## Graphical abstract

**Figure:**
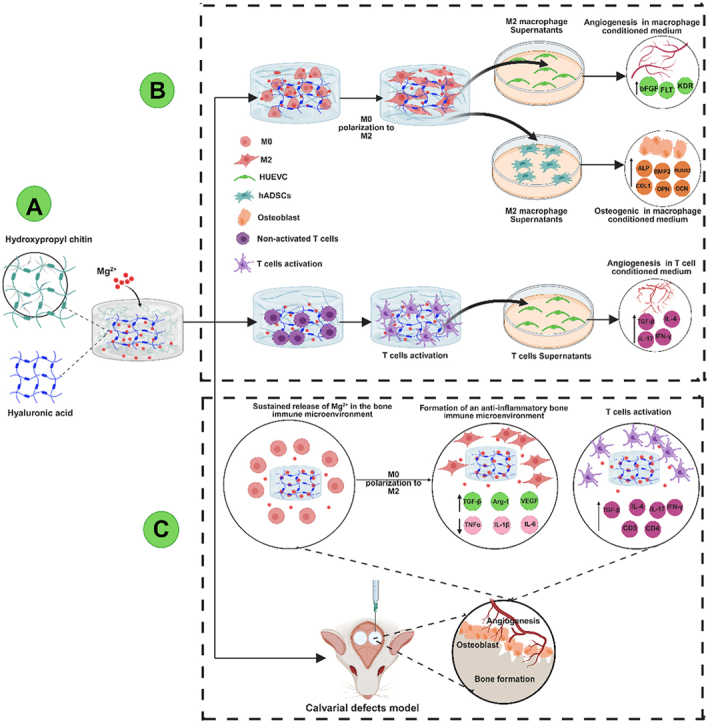

Scheme. (A) Synthesis of nano MgO loaded complex hydrogels. (B) Nano MgO loaded complex hydrogels promotes the polarization of macrophages to type M2 and activates T cells. Collect the supernatant of polarized M2 macrophages to prepare a conditioned medium to promote osteogenesis and angiogenesis, and collect the supernatant of activated T cells to prepare a conditioned medium to promote angiogenesis. (C) Nano MgO loaded complex hydrogels continuously releases magnesium ions at the site of mouse skull defects, activates M2 macrophages and T cells, and forms an anti-inflammatory immune microenvironment that promotes bone repair.

## 1 Introduction

Bone tissue, an essential mineralized component of the human body, has the inherent ability to regenerate and repair itself through the biological activities of bone-related cells. Despite this natural capacity for self-renewal, the repair of large bone defects remains a substantial challenge owing to the limited self-repair capabilities of the bone tissue.

Immune cells, including macrophages and T lymphocytes, are found during all phases of bone repair and modulate bone metabolism by participating in the complex processes of bone remodeling and immune response [1,2]. During bone remodeling, old or damaged osteoclasts and osteoblasts undergo apoptosis; macrophages phagocytose these cells, aiding in the removal of cellular debris and facilitating new bone formation. In addition, macrophages release cytokines, such as interleukins, which can influence both osteoclast and osteoblast activity, affecting bone resorption and formation [3]. Once activated, T cells are able to produce cytokines, including interferon-gamma (IFN-γ) and interleukin-17 (IL-17), which can inhibit osteoblast activity, stimulate osteoclastogenesis, and affect angiogenesis, which is essential for bone development [4,5]. Immunomodulatory T cells (Th2) or suppressive T cells (regulatory T cells) release factors like TGF-β and IL-10, which broadly suppress the activity of activated T cells [6]. Therefore, immune cell dysfunction leads to an imbalance in bone metabolism, resulting in osteoporosis, rheumatoid arthritis, osteoarthritis, and other diseases [7]. This suggests that targeting the immune cells involved in bone metabolism emerges as an effective therapeutic strategy for managing bone diseases.

During bone injury, macrophages are rapidly recruited to the injured area and release active factors that have a significant chemotactic effect on stem cells and promote bone healing. hADSCs are abundant in materials, easy to obtain, have strong in vitro proliferation ability, are easy to culture, have extraction concentrations that are significantly higher than those of MSCs from the bone marrow and umbilical cord, and cause almost no harm to the body [8].

Magnesium, ranking as the fourth most abundant cation in the human body, plays a key role in enzyme and membrane processes, acts as an antagonist of calcium, and plays several structural roles in proteins and nucleic acids [9]. Adequate magnesium supplements are sometimes prescribed to patients with bone fractures or to those undergoing orthopedic surgery to support bone healing and overall recovery, which may enhance the growth and differentiation of osteoblasts. and inhibit excessive osteoclast activity [10]. Moreover, magnesium has anti-inflammatory properties that can be beneficial in reducing inflammation at the location of bone injury by controlling the polarization of macrophages [11]. However, it is still unclear whether magnesium is involved in the regulation of adaptive T lymphocytes.

Existing biomaterials mainly focus on simple bone repair, overlooking the relationship between biomaterials and the immune system, and the role of blood vessels in bone formation. In the past few years, the microenvironment is known to be a key factor in the osteogenesis process [[12], [13], [14]]. On this basis, we aimed to construct a hydrogel that can regulate the immune microenvironment to serve as an ideal bone graft substitute for use in clinical bone defect treatment.

Previously, we created the moldable thermosensitive hydroxypropyl chitin (HPCH) hydrogel, which possesses satisfactory biocompatibility and regenerative capacity, mechanical range (2–8 MPa), and a gelation time of <18 s at 37 °C [[15], [16], [17]]. Hyaluronic acid (HA) has several unique biological properties, including viscoelasticity, regenerative capacity, anti-inflammatory properties, and biocompatibility, that make it an essential component of tissues [[18], [19], [20], [21], [22]]. Therefore, we designed a new thermosensitive hyaluronic acid–hydroxypropyl chitin hydrogel(HPCH@HA) with good affinity, high viscosity, and sustained release of Mg^2+^, and explored its regulatory effect on the bone immune microenvironment. We cultured macrophages and T lymphocytes on HPCH@HA + MgO to examine how Mg^2+^ influence the macrophage inflammatory response and T lymphocyte activation, and then observed the effects of conditioned media on hADSC osteogenic activity and angiogenesis of HUVECs. Finally, a mouse skull defect model was employed to assess the in vivo immunomodulatory response of composite hydrogels. This study demonstrated that a nano MgO loaded complex hydrogels can be employed as a highly effective immunomodulatory biomaterial for bone tissue engineering (**Scheme)**.

## 2 Materials and methods

### 2.1 HPCH@HA + MgO hydrogel preparation

According to our previous research, hydroxypropyl chitin (HPCH) was synthesized in a homogeneous solution as described in the previous work,the average HPCH viscosity was Mw = 414 kDa [23], the molar degree of substitution of hydroxypropyl (MDS) was 0.85, and the degree of acetylation (DA) measured by proton nuclear magnetic resonance (^1^H NMR) was 0.90 [24]. The HPCH powder was sterilized with ethylene oxide and placed in 25 mL glass bottles. Subsequently, 20 mL of deionized water was added to each bottle, stirred magnetically for 15 min under the powder was completely dissolved, and placed in a refrigerator at 4 °C overnight. After the HPCH dispersion was fully swollen, 10 mg of HA (Mw = 10 kDa) [25] (Solarbio, China) powder was added, stirred evenly with a glass rod, placed in an ultrasonic cleaner, and mixed for 15 min. An HPCH–HA solution with a mass ratio of 12:1 was obtained. The HPCH–HA solution and MgO (Sigma, Germany) nanoparticles were then stirred at room temperature for 24 h to form an HPCH@HA + MgO composite hydrogel. The HPCH–HA solution was placed in a −20 °C refrigerator overnight until fully frozen, and then placed in a vacuum freeze dryer and freeze-dried for 24 h to obtain HPCH@HA + MgO powder.

### 2.2 Scanning electron microscopy (SEM)

To study the internal structure of HPCH@HA + MgO hydrogel, All samples were frozen in liquid nitrogen directly for 72 h and dehydrated hydrogel material on a suitable copper grid and sputtered gold/palladium for 60 s. Scanning electron microscope (Hitachi SU8010, Japan) and iXRF EDS system were used to take pictures and preserve them.

### 2.3 Rheological testing

A rheometer with a temperator-controlled water bath system and parallel plate fixture was used to test the rheology of the gel(HAAKE Rheo Stress 6000, Thermo Scientific, German). The testing process was carried out in the linear viscoelastic region of the gel. The hydrogel aqueous solution was placed in the rheometer clamp, and the sample was dynamically scanned by temperature from 4 °C to 37.5 °C, and then dropped from 37.5 °C to 4 °C. The storage modulus (G′) and loss modulus (G ‴) of the sample as a function of temperature and the gaging temperature were recorded.

### 2.4 Mg^2+^ sustained release quantitative detection

We tested the sustained release of Mg^2+^ from the HPCH@HA + MgO hydrogels. HPCH@HA + MgO was immersed in PBS at 37 °C and samples were taken at 1, 3, 5, 7, 9, 11, 13, 15, 17, 19, and 21 days to detect the Mg^2+^ concentration. At each time point, 200 μl of supernatant was collected and 200 μl of PBS was added. After the supernatant was lightly centrifuged, the Mg^2+^ concentration was quantitatively detected by inductively coupled plasma optical emission spectroscopy (ICP–OES) at 37 °C. Each sample was analyzed in triplicate.

### 2.5 Extraction and identification of hADSC

Clean and uncontaminated human subpatellar fat pad tissue blocks were placed in sterile sampling tubes; the procedure was approved by the ethical review board of the Union Hospital Affiliated to Huazhong University of Science and Technology (UHCT-IEC-SOP-016-03-01). The excised tissue was rinsed thoroughly with a 0.9 % sodium chloride solution containing gentamicin sulfate and placed in PBS. Within 30 min, the fat tissue was placed in a sterile culture dish, the fascia and blood vessels were removed, and the fat tissue was cut into pieces of approximately 1 mm^3^ with scissors and placed in a 50 ml centrifuge tube at 25–35 ml/tube, and type I collagenase (Beyotime, China) was added. Tubes were placed in a water bath at 37 °C, centrifuged, digested, and centrifuged, after which the supernatant was discarded and culture medium containing 15 % fetal bovine serum (ExCell Bio, China) was added. Next the sample was transferred to a 10 cm^2^ sterile culture dish, and cultured in an incubator. The medium was changed for the first time after 3 days and thereafter once every 3 days. When cell adherence was 80 %–90 %, cell passage (1:2 passage) was performed. Third-generation, well-grown hADSCs were plated at a density of 1 × 10^5^ cells/well in a 6-well plate. Surface markers of hADSCs were identified using flow cytometry (CytoFLEX, USA): CD34, CD44, CD73, and CD90 (all from BD, USA).

### 2.6 Cell culture and cell-conditioned medium collection

THP-1 cells, human primary T cells CD3^+^ CD4^+^, and HUVECs were purchased from Wuhan Pronocell Co., Ltd. (Pronocell, China). THP-1 human primary T cells were cultured in RPM1640 medium (Gibco, USA) containing 10 % fetal bovine serum (ExCell Bio, China) and HUVECs were cultured in DMEM-F12 medium (Gibco, USA) containing 10 % fetal bovine serum at 37 °C and 5 % CO_2_; the medium was changed every 2 days. Phorbol myristate acetate (MCE, USA) was used to induce THP-1 differentiation into M0 macrophages for 2 days. Third-generation cells were used for all experiments. The hydrogel groups were as follows: control, HPCH@HA, HPCH@HA + MgO (2.5 mg/ml), and HPCH@HA + MgO (5 mg/ml). THP-1 human primary T cells were seeded in a 6-well culture plate at a density of 2 × 10^5^ cells/well and cultured in RPM1640 containing fetal bovine serum for 24 h. The nano MgO loaded complex hydrogels was placed in the upper chamber of a transwell (Corning, USA). THP-1 cells, T cells, and the nano MgO loaded complex hydrogels were further co-cultured for 72 h and then seeded in a 6-well culture plate at a density of 2 × 10^5^ cells per well. Antibody identification by T-cell flow cytometry CD3, CD4 (all from BD, USA). The culture medium was collected after 72 h of culture in RPM1640 containing fetal bovine serum. The collected culture medium was centrifuged at 3000 rpm for 10 min, and the supernatant was collected, stored, and labeled as conditioned medium (CM). nano MgO loaded complex hydrogels.

### 2.7 Live-dead staining and flow cytometry of apoptosis

The hydrogels were divided into four groups: control, HPCH@HA, HPCH@HA + MgO (2.5 mg/ml), and HPCH@HA + MgO (5 mg/ml), which were spread flat on the bottom of the confocal dish; 0.5 × 10^5^ macrophages/well were inoculated on the hydrogels. After 72 h of culture, the cells were fixed with 4 % paraformaldehyde and washed twice with PBS. A certain amount of calcein-AM and PI (Thermol, USA) solution was added to PBS to prepare a mixed solution of a certain concentration. The mixed staining solution was added and incubated at 37 °C for 15 min. Green living cells were observed under a fluorescence microscope at 488 nm excitation, which was then adjusted to 594 nm excitation to observe dead red cells. The fluorescence intensity of each group was recorded, and images were captured using an inverted microscope (Nikon, Japan). An Annexin V-FITC/PI apoptosis detection kit was used to detect apoptosis in the macrophages. The specific steps were as follows: each group of hydrogels was spread on the bottom of a 6-well plate, and 2 × 10^5^ macrophages/well were inoculated on the hydrogel. After 72 h of culture, the 6-well plates were carefully washed with PBS and the cells were collected. According to the instructions of the kit, 200 μl of the above-mentioned staining working solution was added to each tube to resuspend the cells, and incubated at room temperature in the dark for 15 min. After incubation, cells were directly placed in a flow cytometer (CytoFLEX, USA).

### 2.8 CCK8 assay was used to detect the toxicity of hydrogel

The hydrogels were divided into four groups: control group, HPCH@HA, HPCH@HA + MgO (2.5 mg/ml), and HPCH@HA + MgO (5 mg/ml). The hydrogels were spread on 96 well plates, and 0.2 × 10^5^ macrophages/well were inoculated on the hydrogels. After 72 h of culture, 10 μl of CCK-8 (MCE, USA) solution was added to each well. Since the amount of CCK-8 added was relatively small, errors may be caused by the reagent sticking to the well wall. It is recommended to gently shake the culture plate after adding the reagent to help mix it. The 96-well plate was placed in an incubator and incubated for 1–4 h. The absorbance value at 450 nm was measured using an enzyme reader (PerkinElmer, UK).

### 2.9 Macrophage polarization detected by flow cytometry

The four hydrogel groups—control, HPCH@HA, HPCH@HA + MgO (2.5 mg/ml), and HPCH@HA + MgO (5 mg/ml)—were spread onto 6-well plates. After gelation, 1.5 × 10^5^ macrophages/well were inoculated on the hydrogels. After 72 h of co-culture, the cells were collected, washed once with PBS, centrifuged at 300×*g* for 5 min, and washed again. The cells were counted and added to a 1 × 10^6^/mL suspension with a cell staining buffer; 100 μL of cell suspension was added to a 2 mL flow tube for later use. Fluorescent-labeled macrophage marker antibodies against CD80, CD86, CD163,CD206 (all from BD Biosciences, USA) were added according to the recommended dosage in the manual. After mixing, the mixture was placed at 4 °C and incubated in the dark for 30 min. We added an appropriate amount of cell staining buffer to resuspend the cells, centrifuged the cell suspension at 300 g for 5 min, discarded the supernatant, added 0.2 mL of cell staining buffer to resuspend the cells, and analyzed each sample using a Flow cytometer (CytoFLEX, USA).

### 2.10 Cell immunofluorescence and phalloidin staining

The expression levels of CD86 and CD206 in polarized THP-1 macrophages were determined by immunofluorescence. The hydrogels were divided into four groups, as described above, and plated in 6-well plates. After gelation, 1.5 × 10^5^ macrophages/well were inoculated on the hydrogels, and the cells were collected after co-culture for 3 days. Macrophages were fixed with 4 % paraformaldehyde for 15 min, and the membrane was disrupted with 1 % Tron-X. Primary antibodies CD86 (1:50; Novus Biologicals) and CD206 (1:50; Abcam) were used at 4 °C overnight, and then goat anti-rabbit secondary antibody Alexa Fluor 594 (1:200; Abcam) was added for further incubation at ambient temperature for 2 h. We then took 200 μl of the prepared FITC-labeled phalloidin working solution, covered the cells on the coverslip, and incubated at room temperature in the dark for 30 min. The cell nuclei were then stained with DAPI solution for 5 min, and the morphology of the macrophages was observed under a fluorescence microscope (Nikon, Japan). The control group consisted of macrophages that were incubated without the hydrogels.

### 2.11 ELISA analysis

For each of the four hydrogels groups—control, HPCH@HA, HPCH@HA + MgO (2.5 mg/ml), and HPCH@HA + MgO (5 mg/ml) inflammatory factors secreted by macrophages on the nano MgO loaded complex hydrogels were detected using an enzyme-linked immunosorbent assay (ELISA; R&D Systems, USA). After gelation, 1.5 × 10^5^ macrophages/well were inoculated on the hydrogels, and the supernatant was collected after co-culture for 3 days. The levels of TNF-α, IL-1β, IL-6, Arg-1, VEGF, and TGF-β in the supernatant were detected by ELISA kits. Sample preparation, ELISA standard dilution, washing during ELISA, and absorbance values of standards and samples were determined, and standard curves were drawn. The corresponding concentration of the sample was calculated from the absorbance values of the sample and standard curve. T cells were inoculated in the same way, and IFN-γ, IL-17, TGF-β, and IL-4 in the supernatant were detected by ELISA.

### 2.12 Gene expression analysis

Polymerase chain reaction (PCR) was performed to determine gene expression. The hydrogel was spread on the bottom of a 6-well plate. After gelation, 1.5 × 10^5^ macrophages and T lymphocytes were inoculated on the hydrogel, and the cells were collected after 3 days of co-culture. The complete medium was updated every 2 days, and RNA was extracted using the TRIzol reagent (Takara, Japan) according to the manufacturer's instructions. A NanoDrop 1000 spectrophotometer (Bio-Rad) was used to determine the total RNA concentration. The RNA was reverse transcribed into cDNA using a Hifair ® V one-step RT-gDNA digestion SuperMix for qPCR kit (11142ES60, YeaSen China). The expression of related genes TNF-α, IL-1β, IL-6, IL-10, VEGF, TGF-β, IL-4, IL-17, and IFN-γ was detected using a Hieff ® qPCR SYBR Green Master Mix (No Rox) kit (11201ES08 YeaSen, China); a fluorescence quantitative PCR system provided (Bio-Read, USA) real-time quantitative PCR. The four groups of macrophages were used to induce hADSC osteogenesis. The medium was changed every 2 days. After 14 days of intervention, the cells were collected, and PCR was used to detect the osteogenic indicators ALP, BMP2, RUNX2, COLL1, OPN, and OCN. The PCR method was the same as described above. Conditioned medium was used to intervene in HUVECs. The medium was changed every 2 days. After 24 h of intervention, the cells were collected and PCR was used to detect the angiogenic indicators FLT, KDR, and bFGF. The RNA sequences are listed in Table 1.

**Table 1:** RNA primer sequence.

Genes	Sequence
ALP	Forward (5′–3′):CAAATGCCTGGATCCTGTTGAC
	Reverse (5′–3′):TGCACTGGCCATCCATCTC
BMP2	Forward (5′–3′):GTCCCGACAGAACTCAGTGCTATC
	Reverse (5′–3′):CACCCACAACCCTCCACAACC
RUNX2	Forward (5′–3′):CACTGGCGCTGCAACAAGA
	Reverse (5′–3′):CATTCCGGAGCTCAGCAGAATAA
OPN	Forward (5′–3′):ACACATATGATGGCCGAGGTGA
	Reverse (5′–3′):GTGTGAGGTGATGTCCTCGTCTGTA
COLL1	Forward (5′–3′):TAGGGTCTAGACATGTTCAGCTTTG
	Reverse (5′–3′):CGTTCTGTACGCAGGTGATTG
OCN	Forward (5′–3′):CGGTGCAGAGTCCAGCAAA
	Reverse (5′–3′):GGTAGCGCCTGGGTCTCTTC
β-actin	Forward (5′–3′):TGGCACCCAGCACAATGAA
	Reverse (5′–3′):CTAAGTCATAGTCCGCCTAGAAGCA
TNF-α	Forward (5′–3′):CTGCCTGCTGCACTTTGGAG
	Reverse (5′–3′):ACATGGGCTACAGGCTTGTCACT
IL-1β	Forward (5′–3′):CCAGGGACAGGATATGGAGCA
	Reverse (5′–3′):TTCAACACGCAGGACAGGTACAG
IL-6	Forward (5′–3′):AAGCCAGAGCTGTGCAGATGAGTA
	Reverse (5′–3′):TGTCCTGCAGCCACTGGTTC
Arg-1	Forward (5′–3′):ACCTGCCCTTTGCTGACATCC
	Reverse (5′–3′):TCTTGACTTCTGCCACCTTGCC
VEGF	Forward (5′–3′):GCAGGAGCCGCAGTGGTAAG
	Reverse (5′–3′):ACAGCCAAGGTCACAGGAAGC
TGF-β	Forward (5′–3′):GTGAGCCTTGTAGCCTGGATGG
	Reverse (5′–3′):GAGCACCTGGTCAGCAGATGG
IL-10	Forward (5′–3′):CTGCGGAGGTGCCTGAGAATG
	Reverse (5′–3′):AGCCCACGAGGAGGACATAGG
IL-4	Forward (5′–3′):CAAGTAGACAGGCAGGCAAGAC
	Reverse (5′–3′):GTACTCTGGTTGGCTTCCTTCAC
IL-17	Forward (5′–3′):ACTACAACCGATCCACCTCAC
	Reverse (5′–3′):ACTTTGCCTCCCAGATCACAG
IFN-γ	Forward (5′–3′):TTCTTACAACACAAAATCAAATCA
	Reverse (5′–3′):TCAACAAAGCTGATACTCCA

### 2.13 Western blot

After hADSCs were induced to differentiate into osteoblasts with macrophage-conditioned medium for 14 days, cell lysate was prepared using RIPI and protease inhibitors (Beyotime, China) for protein extraction. The protein concentration was determined using a BCA detection kit (Beyotime, China). SDS-PAGE electrophoresis was then performed on a PVDF membrane (Millipore, USA), blocked with blocking buffer (Biosharp, China), and incubated with primary antibodies. OPN (Proteintech China, 22952-1-AP), COLL1 (Proteintech China, 10336-1-AP), OCN (abclone China, A20800), RUNX2 (Affinity China, AF5186), BMP2 (Affinity China, AF5163), ALP (Affinity China, DF6225), and GAPDH (Proteintech China, 60004-1-Ig) were used. Then, the cells were incubated with secondary antibody (Proteintech China, RGAR001) at 4 °C overnight. The images were exposed using a chemiluminescence analyzer (Bio-Rad, USA, 1708370) and analyzed using Image J software.

### 2.14 Transwell assay

In the Transwell migration experiment, hADSC cells were seeded in the upper chamber of a 12-well Transwell chamber at a density of 1 × 10^5^ cells/well per well and cultured with FBS-free and DMEM-F12 medium. The above four different conditioned media were mixed with 10 % FBS DMEM-F12 medium in a ratio of 1:1 to prepare the transwell lower chamber medium, which was added to the lower chamber of a 12-well Transwell plate, cultured for 12 h, and then the upper chamber was removed from the Transwell plate, the cells were fixed with 4 % paraformaldehyde (PFA), and then stained with 1 % crystal violet. Photos were taken with an inverted microscope (NIKON, Japan).

### 2.15 Tube formation test

The above four different conditioned media were mixed with 10 % FBS DMEM-F12 medium in a ratio of 1:1 to prepare fresh culture medium. Matrigel (Corning #356231, USA) was thawed in advance at 4 °C. 10μ of Matrigel was spread on ibidi μ-Slide 3D angiogenesis slide (ibidi, Germany) in each well and placed in a culture dish. The entire culture dish was placed in an incubator and left to stand for about 30 min to wait for the gel to coagulate. A cell suspension with a density of 2 × 10^5^ cells/ml HUVECs was prepared, and 50 μl of HUVECs cell suspension was added to each wel,IL-17 neutralizing antibodies (R&D, MAB13352), IFN-γ neutralizing antibodies (R&D,MAB285). Cover the lid and let it stand. After a period of time, all cells will sink to the surface of Matrigel. After 8 h, an inverted microscope (NIKON, Japan) was used to take pictures, and the length of the tube, the number of rings, the number of nodes, etc. were measured and recorded, and statistical analysis was performed.

### 2.16 ARS staining

The third generation hADSCs were cultured in osteogenic differentiation induction medium (Cyagen, USA) for 14 days or conditioned media, and the medium was changed every 3 days. After induction, they were fixed with 4 % paraformaldehyde. ARS stain (Solarbio.China) was used to stain the mineralized nodules to evaluate their osteogenic differentiation ability. The staining was performed for 30 min according to the instructions, and the cells were observed and photographed under an optical microscope (Nikon, Japan).

### 2.17 ALP staining

The third generation hBMSCs were cultured in osteogenic differentiation induction medium (Cyagen, USA) for 14 days or conditioned media, and the medium was changed every 3 days. After induction, they were fixed with 4 % paraformaldehyde. ALP staining kit (Beyotime.China) was used for staining to evaluate its osteogenic differentiation ability. ALP staining solution was prepared according to the instructions of the kit, and then the cells were incubated in the dark for 30–60 min. Observed and photographed under an optical microscope (Nikon, Japan).

### 2.18 Oil Red O staining

The third generation hADSCs were cultured in adipogenic differentiation induction medium (Cyagen, USA) for 14 days, and the medium was changed every 3 days. After induction, they were fixed with 4 % paraformaldehyde. Oil Red O staining kit (Solarbio.China) was used for staining to evaluate the adipogenic differentiation ability of stem cells. The staining solution was prepared according to the instructions of the kit, and then the cells were incubated in the dark for 30 min. Observed and photographed under an optical microscope (Nikon, Japan).

### 2.19 Alcian blue staining

The third generation hADSCs were cultured in chondrogenic differentiation induction medium (Cyagen, USA) for 14 days, and the medium was changed every 3 days. After induction, they were fixed with 4 % paraformaldehyde. Alcian blue staining (Beyotime.China) was used for staining to evaluate its chondrogenic differentiation ability. Alcian blue staining solution was prepared according to the instructions of the kit, and then the cells were incubated in the dark for 30 min. Observed and photographed under an optical microscope (Nikon, Japan).

### 2.20 Animal experiments

Male adult C57 mice (8 weeks old) were used for relevant experiments in accordance with the guidelines of the Animal Care and Use Committee of Huazhong University of Science and Technology (IACUC Number: 4008) and the National Research Council's Guide for Care and Use of Laboratory Animals. The animals had ad libitum access to food and water. To evaluate the ability of the nano MgO loaded complex hydrogels to induce bone regeneration, the mice were anesthetized with sevoflurane. All the mice were shaved and disinfected prior to surgery. A 3 mm diameter craniotomy defect was created on the skull on both sides of the sagittal suture of each C57 mouse. Subsequently, the magnesium-ion porous hydrogel was implanted into the defect (n = 5 per group), the incision was surgically sutured, and penicillin was injected subcutaneously after suturing. The blank group was not implanted with hydrogel and served as the control group. C57 mice were sacrificed 8 weeks later, and specimens were collected for micro computed tomography (microCT) and histological analyses.

MicroCT (SKYSCAN1076, Belgium) was used to scan the bone volume of each group, and the skull was reconstructed three-dimensionally. Bone volume, bone mineral density (BMD), and bone volume fraction (BV/TV) were analyzed using the SkyScan software (Bruker, Germany). The collected skull samples were fixed in 10 % formalin for 24 h, decalcified with EDTA solution for 2 weeks, and dehydrated with different concentrations of ethanol. The dehydrated samples were immersed in pure xylene solution and embedded in paraffin. Histological analysis was divided into three parts, and the specimens were labeled with CD86, CD206, CD3, and CD4 antibodies (all from Proteintech, China) for immunofluorescence staining to detect changes in macrophage phenotype and T cell activation parameters in vivo. HE, Goldner, and Masson staining were performed according to the manufacturer's instructions for histological analysis of new bone tissue. Immunohistochemical staining of BMP2 (Affinity, China), OPN (Affinity, China), Arg-1 (Proteintech, China), TNF-α(Proteintech, China), VEGF (Affinity, China), and FLT (Affinity, China) was performed to observe bone tissue, macrophage polarization, and vascularization.

### 2.21 Transcriptome sequencing

We co-cultured the nano MgO loaded complex hydrogels (5 mg/ml group) with THP-1-derived M0 macrophages. The control group did not contain the nano MgO loaded complex hydrogels. Subsequently, the cells were collected for transcriptomic analysis. The supernatant of the co-culture of nano MgO loaded complex hydrogel (5 mg/ml group) and M0 macrophages was collected to prepare the conditioned medium for the intervention of ADSC. Subsequently, cells were collected for transcriptomic analysis. total RNA was extracted from the tissue samples. The concentration and purity of the extracted RNA were detected using Nanodrop2000. The integrity of RNA was detected by agarose gel electrophoresis, and the RQN value was determined by Agilent5300. For a single library construction, the total RNA amount is required to be 1 μg, the concentration ≥30 ng/μL, RQN >6.5, and OD260/280 ranging from 1.8 to 2.2. The final database was obtained through the following steps: enrichment of mRNA with Oligo dT, fragmentation of mRNA, reverse synthesis of cDNA, connection of adapter, fragment screening and library enrichment, sequencing on the machine. Transcriptome sequencing and analysis were completed at Majorbio Bio-pharma Biotechnology Co., Ltd. (Shanghai, China) by using Illumina HiSeq X10 (Illumina, San Diego, California). Use the TopHat (http://tophat.cbcb.umd.edu/, Version 2.1.1) of each sample software will Clean Reads the specified reference genome Homo_sapiiens v.G RCh38 (http://asia.ensembl.org/Homo_sapiiens/Info/Index) for sequence alignment, The comparison rates range from 98.33 % to 98.88 %. Then, map the clean data to the reference genome. In addition, in the United States majorbio cloud platform (https://cloud.majorbio.com/page/tools.html) for the volcano plot, heatmap, GO enrichment, Kegg enrichment, Reactome enrichment analysis.

### 2.22 Statistical analysis

A Two-way test was used for comparison between the two groups, with quantitative indicators expressed as the mean ± standard deviation ('x ± s). One-way ANOVA was used for the comparison of means among groups, with the Bonferroni test used for post hoc pairwise comparison.

## 3 Results

### 3.1 Characterization of HPCH@HA + MgO hydrogels

**Fig. 1:**
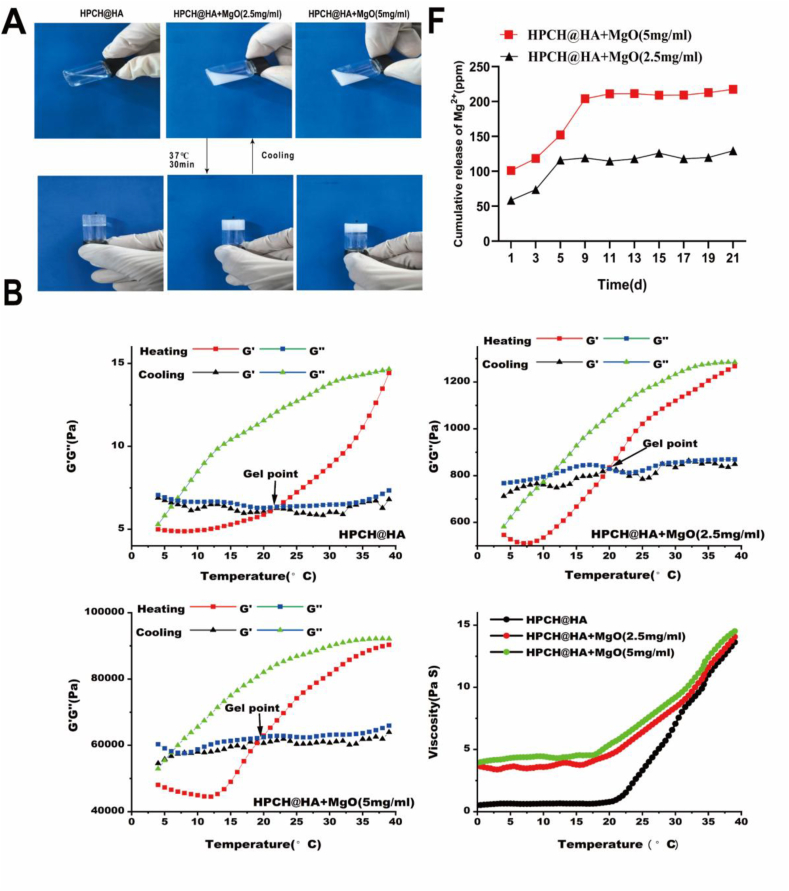

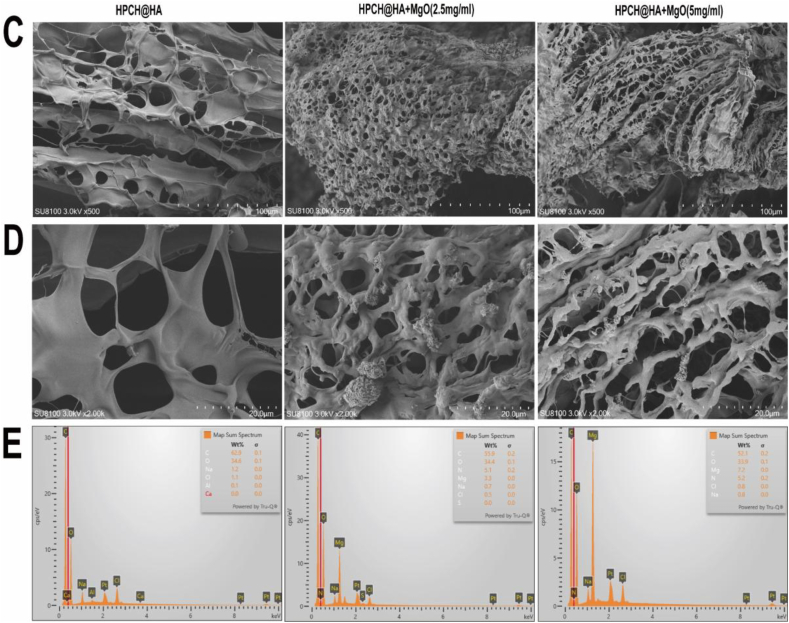
Characterization of nano MgO loaded complex hydrogels. (A) The composite hydrogel quickly solidified into a gel at 37 °C and became liquid at 4 °C. (B) solution-to-gel transition temperature and the values of G'and G’’. (C、D) The morphology, internal structure, and Mg^2+^ distribution of the composite hydrogel were observed by SEM (scale bar = 100 μm、20 μm). (E) EDS analysis confirmed the presence of a large amount of Mg in the microscopic microstructure of the composite hydrogel. (F) The cumulative release of Mg^2+^ from the composite hydrogel in PBS buffer (without Mg^2+^) was measured in vitro at 37 °C.

Gelation of the hydrogels was observed. At 4 °C, the hydrogel was in a uniform liquid state, but it quickly gelled at 37 °C, confirming that our composite hydrogel has a good gelation effect (Fig. 1A). As shown in (Fig. 1B), during the heating process starting at 4 °C, the storage modulus (G′) was lower than the loss modulus (G″) for HPCH@HA, HPCH@HA + MgO (2.5 mg/ml), and HPCH@HA + MgO (5 mg/ml), indicating a solution state. With increasing temperature, the points where G′ = G'′, representing the solution-to-gel transition, occurred at 21 °C, 19.5 °C, and 19 °C, respectively.As the temperature further increased, G′>G'′ and the viscosity rose rapidly. Moreover, the addition of MgO did not significantly alter the solution-to-gel transition temperature or the values of G'and G′′. Upon cooling, both HPCH@HA、HPCH@HA + MgO (2.5 mg/ml) and HPCH@HA + MgO (5 mg/ml) reverted from the gel state back to the solution state. Next, we observed the surface structures of the freeze-dried hydrogels using scanning electron microscopy (SEM). The HPCH@HA hydrogel had uniform pores, and MgO nanoparticles were uniformly loaded onto its surface. EDS also showed that Mg was significantly increased in HPCH@HA + MgO, whereas no Mg was detected in the HPCH@HA hydrogel (Fig.1C–E). Subsequently, we tested the sustained release of Mg^2+^ from the HPCH@HA + MgO hydrogels. We soaked HPCH@HA + MgO in PBS and took samples at 1, 3, 5, 7, 9, 11, 13, 15, 17, 19, and 21 days to test the Mg^2+^ concentration. The results showed that the release increased rapidly on day 1. Subsequently, the release slowed, forming a flat curve until day 21; the HPCH@HA + MgO (2.5 mg/ml) group increased to 100 ppm and the HPCH@HA + MgO (5 mg/ml) group increased to 200 ppm, and then remained stable until day 21 (Fig. 1F). Therefore, it is expected that the composite hydrogel can target the immune microenvironment of bone tissue to regulate the release of Mg^2+^ and induce bone regeneration.

### 3.2 Hydrogel toxicity and biocompatibility

**Fig. 2:**
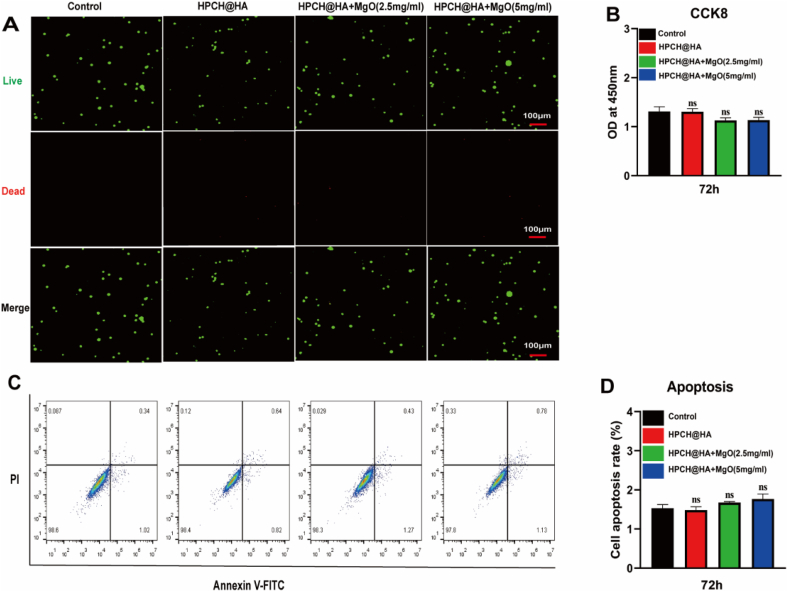
Cytotoxicity and biocompatibility of composite hydrogels. (A) After macrophages was inoculated on the hydrogel for 72 h, live-dead fluorescence staining was performed and images were taken using a confocal microscope. Scale bar = 100 μm. (B) After THP-1 was inoculated on the hydrogel for 72 h, CCK8 was used to detect the cytotoxicity of the hydrogel. (C, D) After macrophages was inoculated on the hydrogel for 72 h, flow cytometry apoptosis analysis was performed. ns: nonsignificant.

In view of the good adhesion and loose porous structure of HPCH@HA, which are conducive to cell adhesion and growth, we inoculated macrophages onto the nano MgO loaded complex hydrogels. First, the survival ability of macrophages co-cultured with the composite hydrogels was studied by live/dead cell staining. Macrophages without any material were used as the controls. After 72 h of coculture, there was no significant difference in cell survival between the hydrogel and control groups (Fig. 2A). We then used the CCK8 to detect cell hydrogel toxicity. Compared with the control group, there was no significant difference in cell proliferation in each group, indicating that the cell viability was good and that the composite hydrogel had better cell biocompatibility (Fig. 2B). Finally, flow cytometry was used to verify the biocompatibility of the composite hydrogel. Compared with the control group, there was basically no death in the composite hydrogel group (Fig. 2C and 2D). These results show that the prepared hydrogel is nontoxic, biocompatible, and conducive to cell growth.

### 3.3 hADSC identification and three-line differentiation

**Fig. 3:**
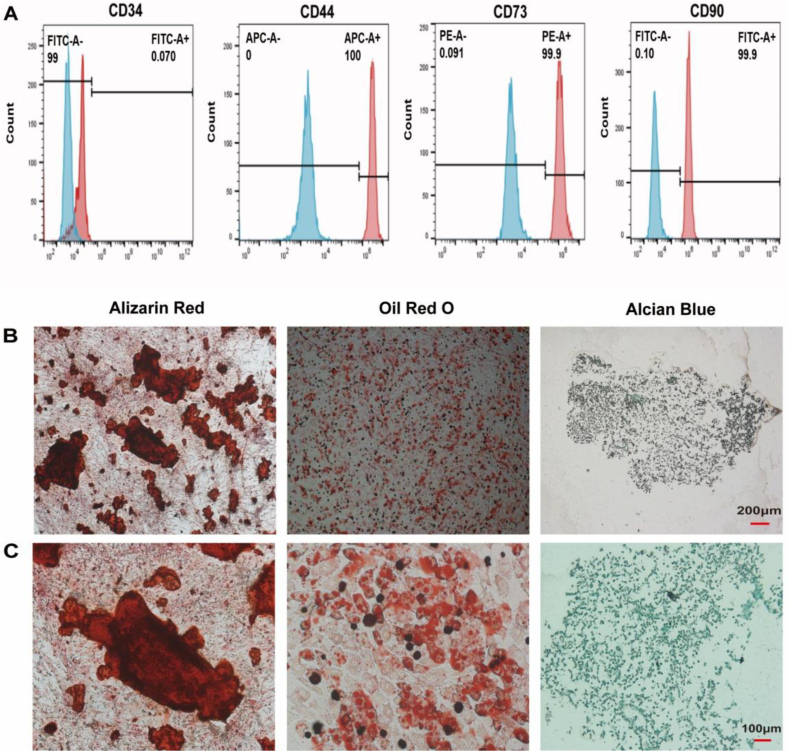
hADSC identification and three-line differentiation. (A) Flow cytometry detection of hADSC surface markers CD34, CD44, CD73, and CD90. (B, C) hADSC osteogenic, adipogenic, and chondrogenic induction for 14 days was followed by ARS, Oil Red O staining, and Alcian Blue staining (scale bar = 200 μm, 100 μm).

We collected third-generation hADSCs that grew well and used flow cytometry to identify them. The results showed that CD34 was not expressed on the surface of the hADSCs, but CD44, CD73, and CD90 were highly expressed (Fig. 3A), indicating that the extracted cells met the surface characteristics of hADSCs. Since hADSCs have a strong differentiation potential, we further identified the osteogenic, adipogenic, and chondrogenic differentiation abilities of hADSCs. Third generation hADSCs that grew well were planted in 6-well plates, and cultured in osteogenic, adipogenic and chondrogenic culture media for 14 days. After the induction of differentiation is completed,ARS, Oil red O and Alcian blue staining were performed to evaluate the differentiation potential of stem cells. The staining results showed that the hADSCs had excellent tri-lineage differentiation abilities (Fig. 3B).

### 3.4 Effects of HPCH@HA + MgO hydrogel on macrophages polarization

**Fig. 4:**
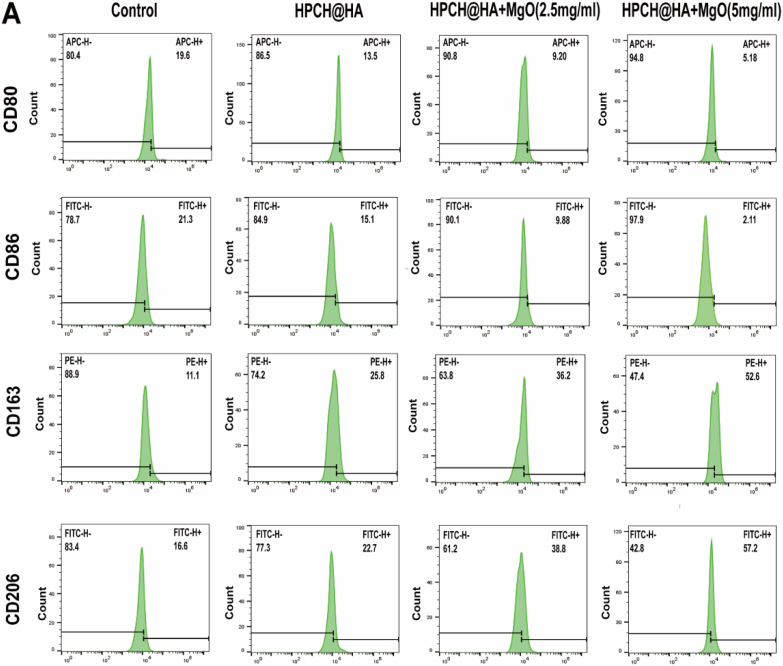

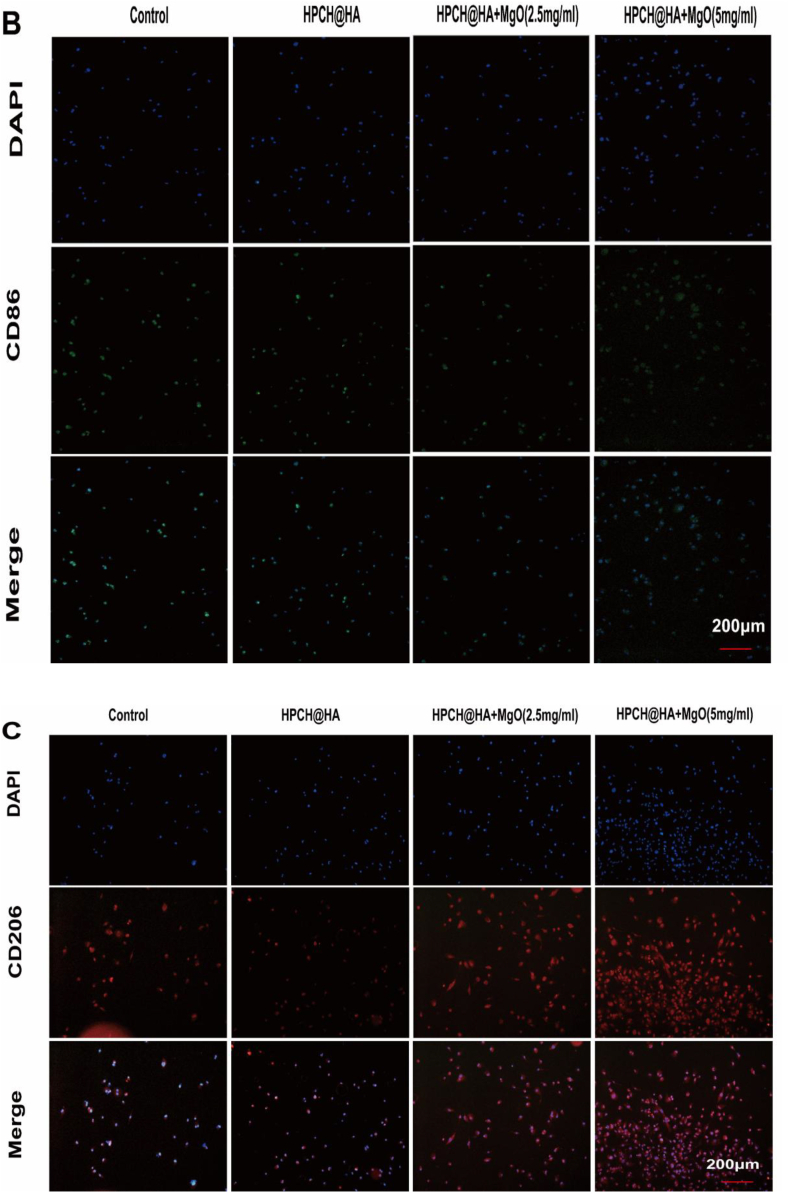

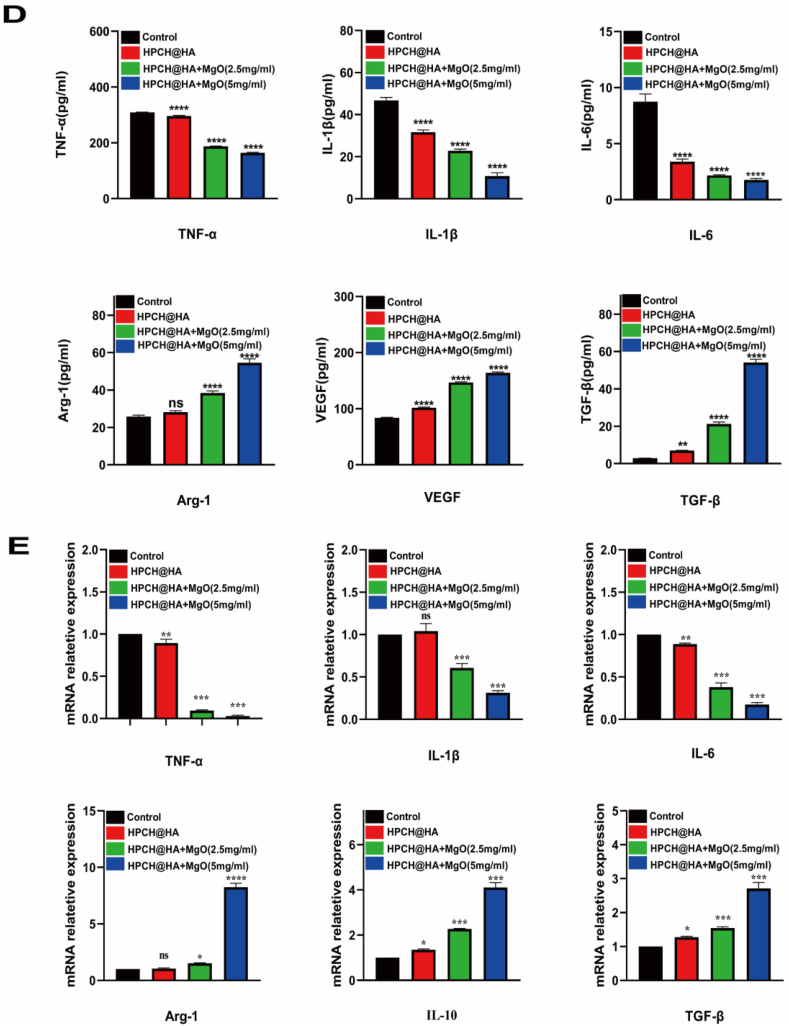
Effects of HPCH@HA + MgO hydrogel on polarization of macrophages. (A) Flow cytometry results of THP-1 cells inoculated in magnesium composite hydrogels for 72 h, showing the ratio of M1 surface markers (CD80, CD86) and M2 surface markers (CD163, CD206). (B, C) Immunofluorescence results of CD86 and CD206 of M1 and M2 macrophage phenotypes in each group. (D) ELISA kits were used to detect the expression of cytokines secreted by THP-1 cells macrophages, including TNF-α, IL-6, IL-1β, Arg-1, VEGF, and TGF-β. (E) Relative expression of mRNA of macrophage surface markers including TNF-α, IL-6, IL-1β, Arg-1, IL-10, and TGF-β. ns: nonsignificant, ∗P < 0.05, ∗∗P < 0.01, ∗∗∗P < 0.001, and ∗∗∗∗P < 0.0001.

Flow cytometry and immunofluorescence staining were used to evaluate the effect of 0 on macrophage polarization. THP-1-induced differentiated macrophages were inoculated onto the composite hydrogel and cultured for 72 h, and flow cytometry was used to determine the ratio of M1/M2 macrophage polarization. Our flow cytometry data showed that the positivity rates of the M1 surface markers CD80 and CD86 of macrophages inoculated on the surface of the HPCH@HA + MgO hydrogel were 9.29 %, 5.18 %, 9.88 %, and 2.11 %, respectively. This was significantly lower than those in the control and HPCH@HA groups. At the same time, we also detected the M2 surface markers CD163 and CD206. The proportions of CD163 and CD206 positive cells in the HPCH@HA + MgO hydrogel groups were 36.2 %, 52.6 %, 38.8 %, and 57.2 %, respectively (Fig. 4A). Next, immunofluorescence was used to detect representative markers of M1 macrophages CD86, and M2 macrophages CD206. Compared with the control and HPCH@HA groups, the HPCH@HA + MgO group expressed a small amount of CD86, and the expression of CD206 was significantly increased (Fig. 4B and 4C). The results showed that the controllable sustained release of Mg2+ from the composite hydrogel promoted the polarization of macrophages to the M2 phenotype. Therefore, the HPCH@HA + MgO hydrogel group induced more M2 phenotypes and fewer M1 phenotypes, and the ratio of M2/M1 macrophages significantly increased.

THP-1-induced differentiated macrophages were inoculated onto the composite hydrogel and cultured for 72 h, and the cell supernatant was collected. ELISA was used to detect the concentration of inflammatory factors secreted by the cells. Our data showed that compared with the control and HPCH@HA groups, the HPCH@HA + MgO group expressed more anti-inflammatory factors (Arg-1, VEGF, and TGF-β) and less pro-inflammatory factors (TNF-α, IL-6, and IL-1β) [Fig. 4D]. In addition, qRT–PCR was used to detect the anti-inflammatory genes Arg-1, IL-10, and TGF-β expressed by M2 and the pro-inflammatory genes TNF-α, IL-6, and IL-1β expressed by M1 (Fig. 4E). Compared with the control and HPCH@HA groups, the pro-inflammatory genes expressed by M1 decreased significantly, whereas the anti-inflammatory genes expressed by M2 increased significantly. These results indicate that the HPCH/HA + MgO hydrogel promotes the polarization of THP-1 macrophages to M2.

### 3.5 Effect of macrophage-conditioned media on angiogenesis of HUVECs

**Fig. 5:**
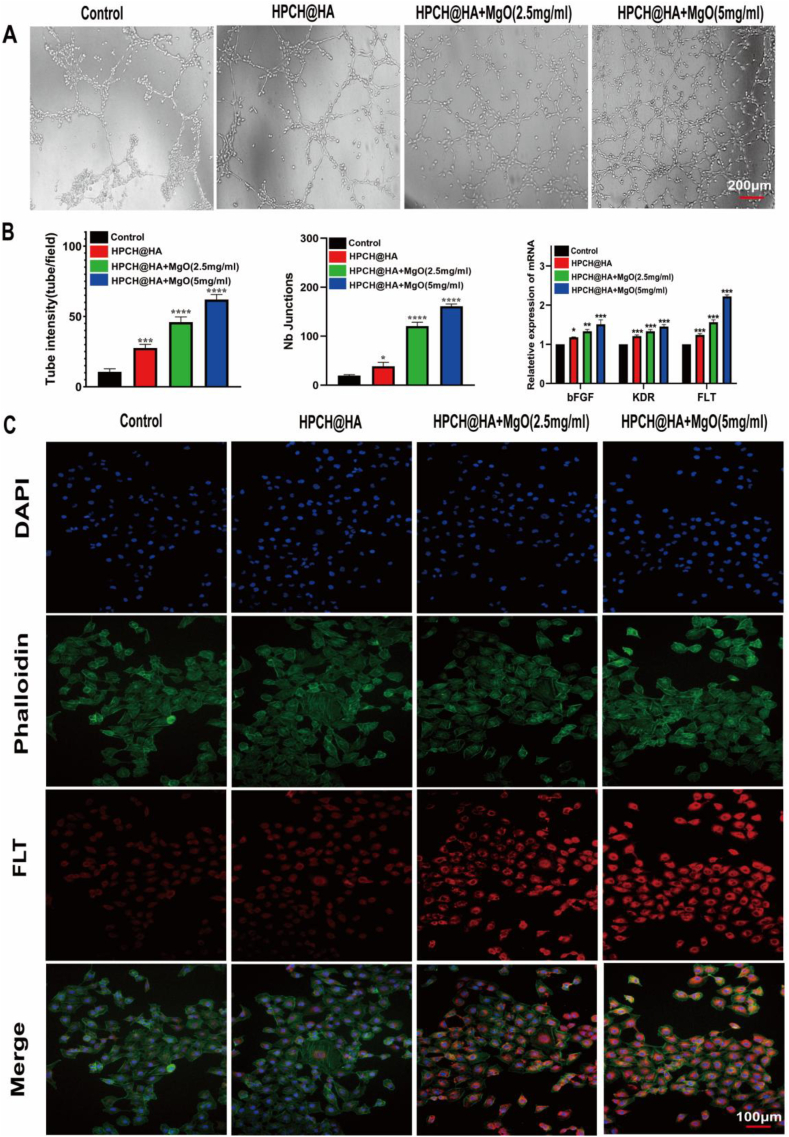
Effect of macrophage conditioned medium on angiogenesis of HUVECs. (A) Angiogenesis of HUVECs cultured with different conditioned media, Scale bar = 200 μm. (B) Expression of bFGF, FLT, and KDR genes in HUVECs cultured with different conditioned media. (C) Immunostaining of FLT and phalloidin cytoskeleton staining in HUVECs cultured with different conditioned media, Scale bar = 100 μm. ns: nonsignificant, ∗P < 0.05, ∗∗P < 0.01, ∗∗∗P < 0.001, and ∗∗∗∗P < 0.0001.

Next, we used macrophage-conditioned media extracted from different groups to observe its effect on HUVECs angiogenesis. We detected the gene expression of several key factors involved in HUVECs angiogenesis, including bFGF, FLT, and KDR. Compared with the control and HPCH@HA groups, the angiogenic gene expression of the conditioned medium of the HPCH@HA + MgO group increased significantly. No changes were observed between the control and HPCH@HA groups (Fig. 5B). Simultaneously, we used immunofluorescence to detect the effects of different groups of conditioned media on FLT fluorescence intensity. Similarly, compared with the control and HPCH@HA groups, the fluorescence intensity of FLT in the conditioned medium of the HPCH@HA + MgO group was the strongest. The fluorescence intensities of the control and HPCH@HA groups were low (Fig. 5C). Therefore, different groups of conditioned media can promote HUVECs angiogenesis, but compared with the control and HPCH@HA groups, the HPCH@HA + MgO group had a stronger vascular ability, greater vascular network density, and more vascular nodes (Fig. 5A and 5B). These data show that HPCH@HA + MgO-conditioned medium can promote HUVECs vascularization. The bone immune environment simulated by the HPCH@HA + MgO hydrogel was conducive to angiogenesis.

### 3.6 Effect of macrophage-conditioned media on osteogenic differentiation of hADSCs

**Fig. 6:**
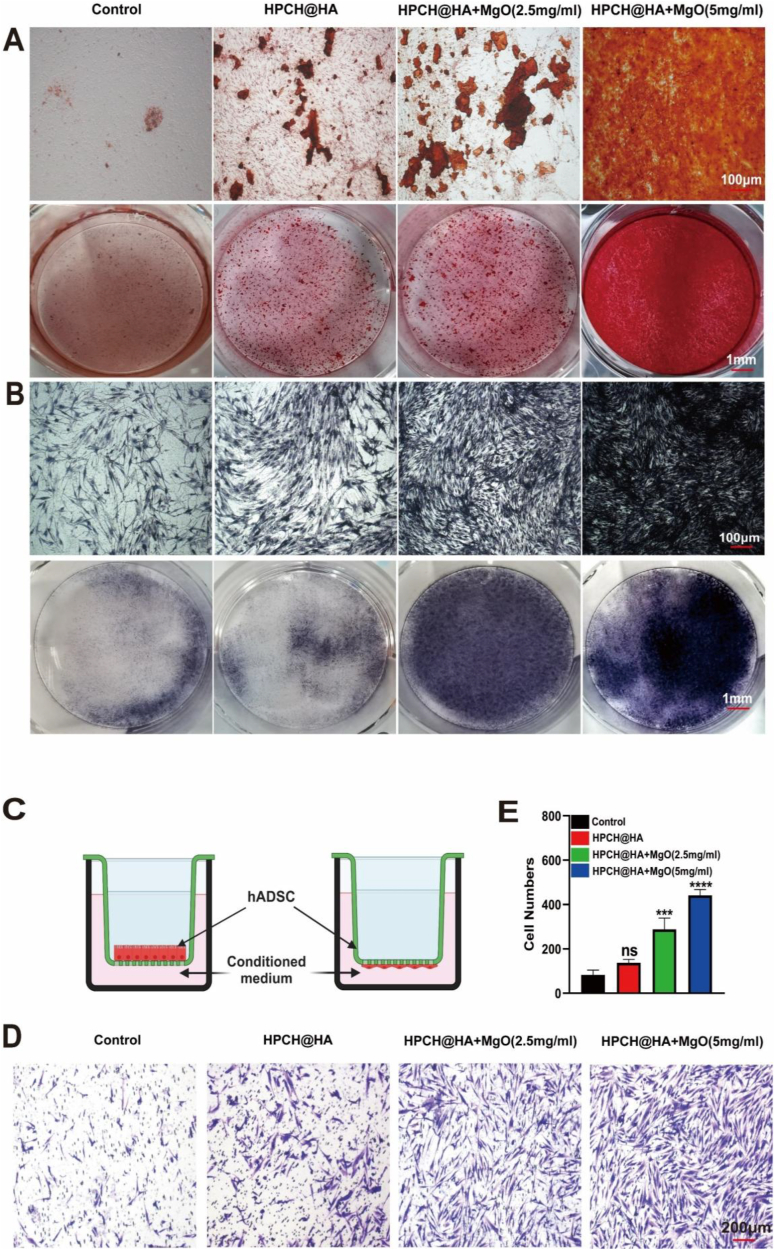

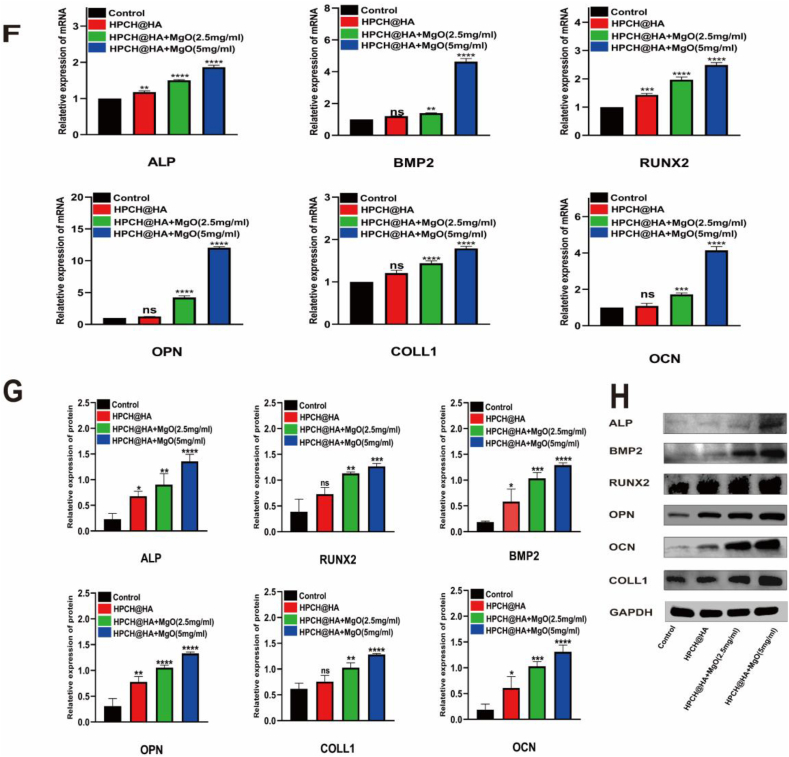
Effect of macrophage-conditioned media on osteogenic differentiation of hADSCs. (A) hADSCs were osteogenically induced with different macrophage-conditioned media for 14 days, and the osteogenic effect was evaluated by ARS assay. (B) hADSCs were osteogenically induced with different macrophage-conditioned media for 14 days, and the osteogenic effect was evaluated by ALP assay. Scale bar = 100 μm. (C, D,E) Transwell assay to evaluate the chemotactic activity and cell number of hADSCs with different macrophage-conditioned media. Scale bar = 200 μm. (F) qPCR detection of the relative mRNA expression of hADSCs after osteogenic induction with different macrophage-conditioned media for 14 days, including ALP, BMP2, RUNX2, OPN, OCN, and COLL1. (G, H) Western blot detection of the relative protein expression of hADSCs after osteogenic induction with different macrophage-conditioned media for 14 days, including ALP, BMP2, RUNX2, OPN, OCN, and COLL1. Scale bar = 100 μm. ns: nonsignificant, ∗P < 0.05, ∗∗P < 0.01, ∗∗∗P < 0.001, and ∗∗∗∗P < 0.0001.

To verify the osteoimmunomodulatory effect of the HPCH@HA + MgO hydrogel, we first induced osteogenic differentiation of hADSCs in different groups of conditioned media. After 14 days of osteogenesis, ARS and ALP staining were performed to identify the osteogenic ability. The results showed that the conditioned medium of each group induced osteogenic differentiation, but that the HPCH/HA + MgO group had the most mineralized nodules and darkest ALP staining (Fig. 6A and 6B). We then performed PCR and western blotting to verify the expression of several key osteogenic factors (ALP, BMP2, RUNX2, OPN, OCN, and COLL1) during osteogenic differentiation. The results showed that the mRNA and protein levels of the osteogenic factors in the control and HPCH@HA groups were unchanged, whereas the mRNA and protein expression of the osteogenic factors in the HPCH@HA + MgO group increased significantly (Fig. 6F–H). We observed the effect of macrophage-conditioned media extracted from different groups on hADSC migration. Transwell dishes were used for the observations. After 24 h, the cells in each group were stained with crystal violet blue. The results showed that each group could promote hADSC migration, but the number of migrated cells in the HPCH@HA + MgO group increased most significantly (Fig. 6C–E). The bone immune environment induced by the HPCH@HA + MgO hydrogel was conducive to the migration, osteogenic differentiation, and mineralization of hADSC, as well as the expression of osteogenic-related genes.

### 3.7 Effects of HPCH@HA + MgO hydrogel on skull defect repair in vivo

**Fig. 7:**
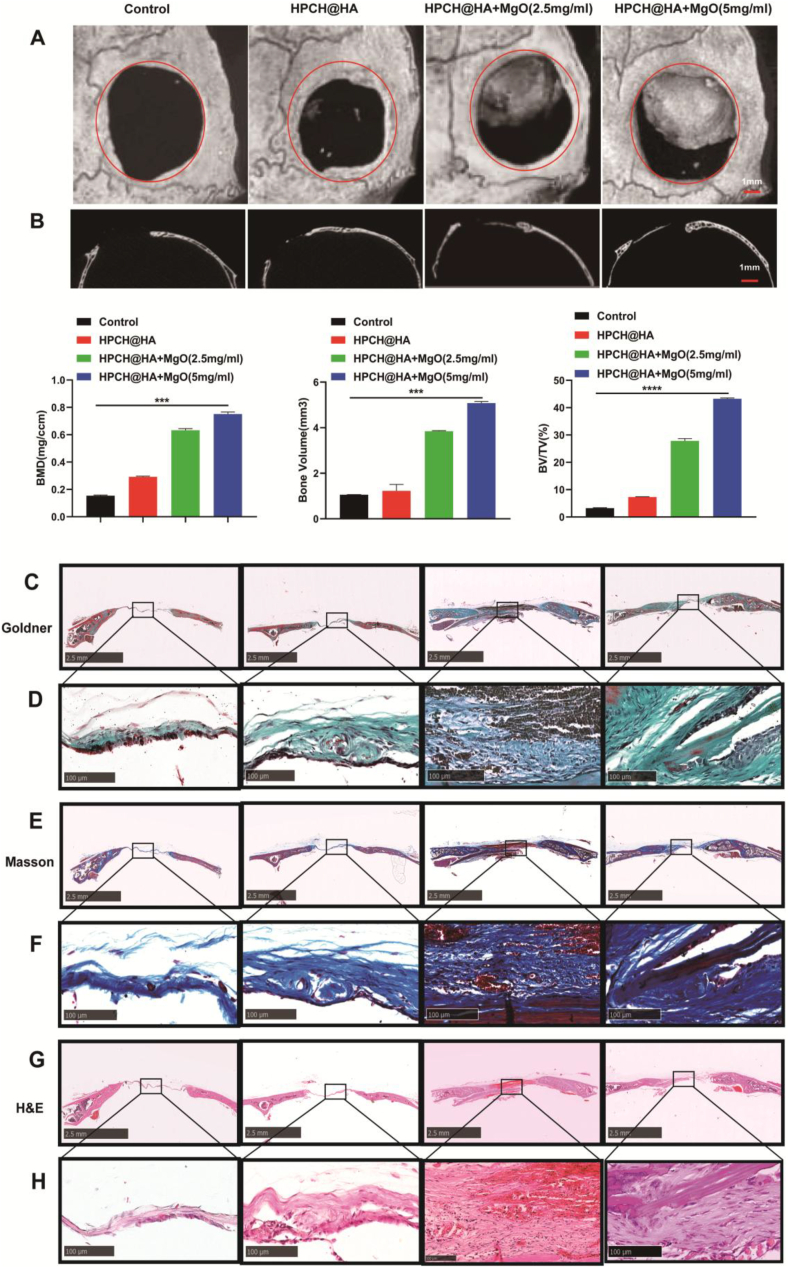
Bone regeneration in the skull bone defect model. (A, B) MicroCT evaluation of bone defect mineralization 8 weeks after surgery, formed during bone defect healing. (C, D) Goldner staining of skull defect 8 weeks after nano MgO loaded complex hydrogels implantation (C shows low-power image, scale bar = 2.5 mm; D shows high-power image, scale bar = 100 μm). (E, F) Masson staining of skull defect 8 weeks after nano MgO loaded complex hydrogels implantation (E shows low-power image, scale bar = 2.5 mm; F shows high-power image, scale bar = 100 μm). (G, H) H&E staining of skull defect 8 weeks after nano MgO loaded complex hydrogels implantation (G shows low-power image, scale bar = 2.5 mm; H shows high-power image, scale bar = 100 μm).

We constructed a mouse skull defect model to observe the effect of HPCH@HA + MgO hydrogel on osteogenesis. At 8 weeks after surgery, we investigated the ability of the composite hydrogel to repair critical bone defects in the skull. MicroCT scanning was used to reconstruct the skull and analyze the new bone (Fig. 7A). The results showed that new bone was formed in each group, but that the quantity and quality of the new bone varied greatly among the four groups. At the same time, the BV/TV, bone volume, and BMD of each group were measured (Fig. 7B). The osteogenic capacity of the HPCH@HA + MgO group was significantly higher than those of the other two groups. To further evaluate the osteogenic capacity of the composite hydrogel, HE, Goldner, and Masson staining were performed on the new bone tissue. Goldner trichrome staining showed that there was only a small number of osteoids in the defects treated with the control and HPCH@HA groups, whereas the HPCH@HA + MgO group showed more mature lamellar bone formation (Fig. 7C and 7D). Masson staining showed similar results, with a large amount of mature fibrous tissue formed in the HPCH@HA + MgO hydrogel, but only a small amount of fibrous tissue formed in the other two groups (Fig. 7E and 7F). In the control and HPCH@HA groups, HE staining revealed only a small amount of new bone. Large lamellar bones were visible in the HPCH@HA + MgO group 8 weeks after surgery. The type I collagen hydrogel group exhibited relatively low osteogenesis (Fig. 7G and 7H). Therefore, our results indicate that the bone immune microenvironment induced by HPCH@HA + MgO in vivo promotes new bone formation.

### 3.8 In vivo immunofluorescence and immunohistochemical staining at 3 days

**Fig. 8:**
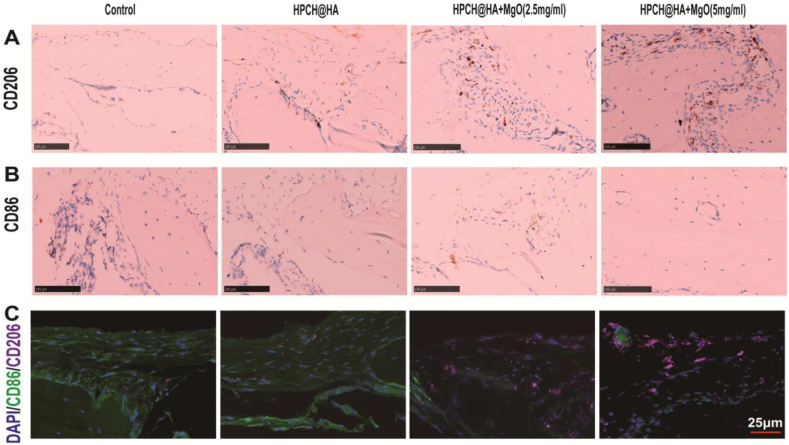
Immunohistochemical fluorescence and immunohistochemical staining of skull defects at 3 days after nano MgO loaded complex hydrogels implantation. (A, B) Immunohistochemical staining of M1 marker CD86 and M2 marker CD206. (C) Immunohistochemical fluorescence staining of M1 marker CD86 and M2 marker CD206. Scale bar = 100 μm. Scale bar = 25 μm.

We evaluated in vivo immune regulation in the bone defect model at 3 days, including immunohistochemical and immunofluorescence staining of macrophages of different phenotypes. Immunohistochemical staining of tissue sections from each group was performed 3 days after surgery. Immunohistochemical results shows that the HPCH@HA + MgO group had the most M2 macrophages CD206 and the least M1 macrophages CD86, indicating a lower inflammatory response (Fig. 8A and 8B). In addition, immunofluorescence staining also demostrates proof the CD206 index in the HPCH@HA + MgO group was significantly increased, and CD86 was decreased, indicating that the proportion of M2 macrophages in the nano MgO loaded complex hydrogels group gradually increase, This indicates that on the 3 day after the inflammatory stage of bone repair, the nano MgO loaded complex hydrogel begins to promote bone repair (Fig. 8C).

### 3.9 In vivo immunofluorescence and immunohistochemical staining at 8 weeks

**Fig. 9:**
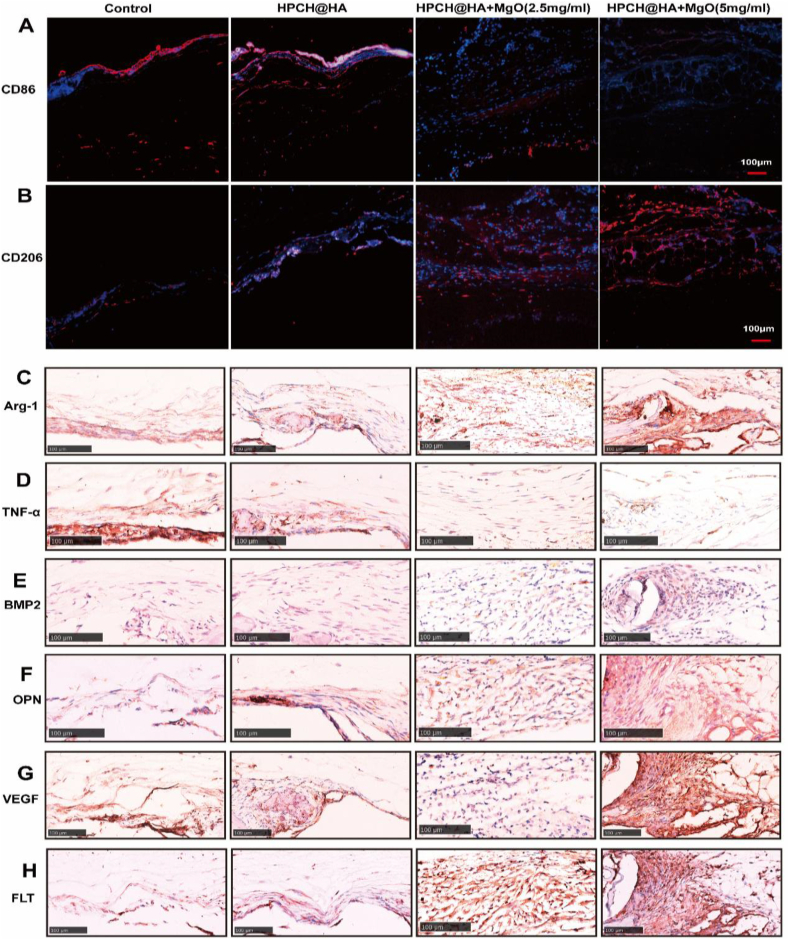
Immunohistochemical fluorescence and immunohistochemical staining of skull defects 8 weeks after nano MgO loaded complex hydrogels implantation. (A, B) Immunohistochemical fluorescence staining of M1 marker CD86 and M2 marker CD206. (C, D) Immunohistochemical staining of M1 macrophage secretion factor TNF-α and M2 macrophage secretion factor Arg-1. (E, F) Immunohistochemical staining of osteogenic markers BMP2 and OPN. (G, H) Immunohistochemical staining of angiogenic markers VEGF and FLT. Scale bar = 100 μm.

We evaluated in vivo immune regulation in the bone defect model, including immunohistochemical staining of inflammatory, osteogenic, and angiogenic factors, and immunofluorescence staining of macrophages of different phenotypes. Immunohistochemical staining of tissue sections from each group was performed 8 weeks after surgery. The HPCH@HA + MgO group had the most M2 macrophages and the least M1 macrophages, indicating a lower inflammatory response (Fig. 9A and 9B). In addition, the Arg-1 index in the HPCH@HA + MgO group was significantly increased, and TNF-α was significantly decreased, indicating that the proportion of M2 macrophages in the nano MgO loaded complex hydrogels group was dominant, which was beneficial to bone healing (Fig. 9C and 9D). The expression trends of the osteogenic factors BMP2 and OPN remained basically unchanged, but were significantly increased in the HPCH@HA + MgO group (Fig. 9E and 9F). Similarly, the angiogenic factors VEGF and FLT were significantly increased in the HPCH@HA + MgO group (Fig. 9G and 9H). These results indicate that the bone immune microenvironment induced by HPCH@HA + MgO was conducive to the polarization of M2 macrophages, forming an anti-inflammatory, osteogenic, and angiogenic immune microenvironment.

### 3.10 Effects of HPCH@HA + MgO hydrogel on T cell activation

**Fig. 10:**
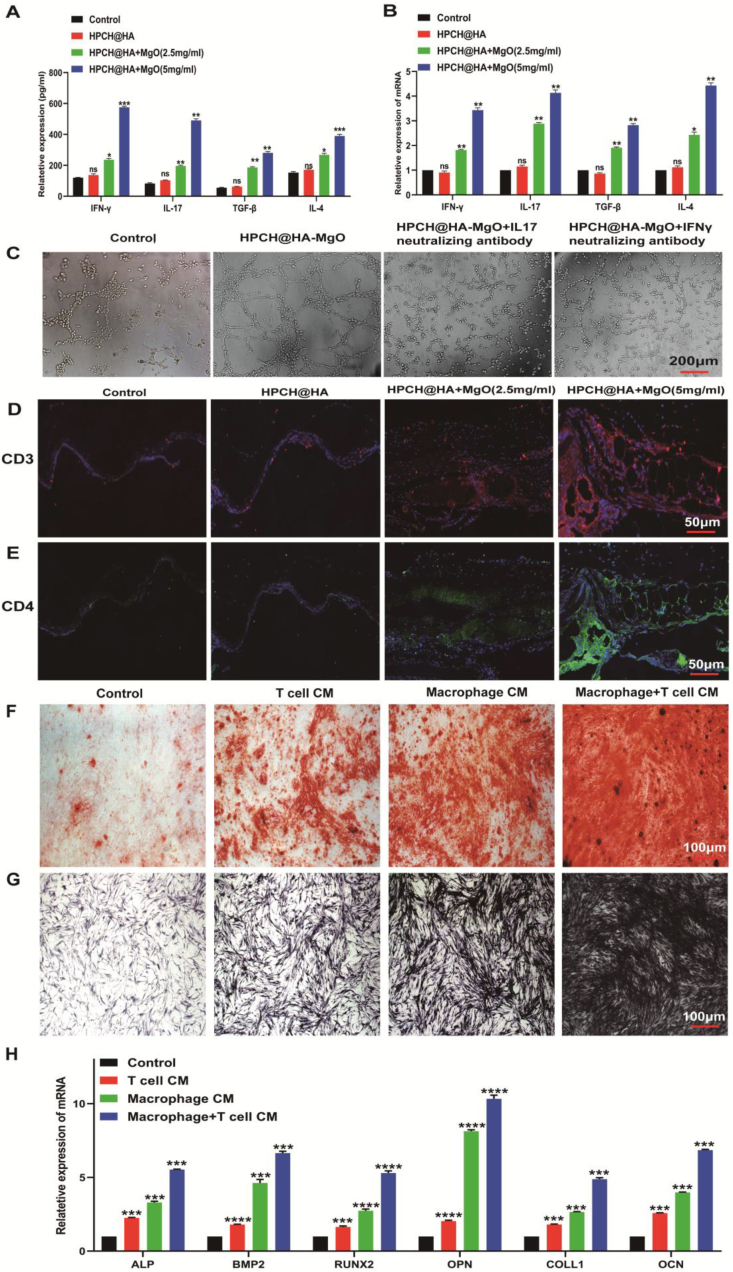
Effect of nano MgO loaded complex hydrogels on T cells. (A) After T lymphocytes were inoculated in nano MgO loaded complex hydrogels and cultured for 72 h, ELISA kit was used to detect the expression of cytokines TGF-β, IL-4, IL-17, and IFN-γ secreted by activated T cells. (B) After T lymphocytes were inoculated in nano MgO loaded complex hydrogels and cultured for 72 h, PCR was used to detect the expression of mRNA of activated T lymphocytes, including TGF-β, IL-4, IL-17, and IFN-γ. (C) Angiogenesis of HUVECs cultured with different conditioned media of T lymphocytes and intervened with IL-17 and IFN-γ neutralizing antibodies. Scale bar = 200 μm. (D, E) Immunohistochemistry was performed on the skull defect 8 weeks after nano MgO loaded complex hydrogels implantation to identify T lymphocyte activation markers CD3 and CD4.Scale bar = 50 μm (F) hADSCs were osteogenically induced with different conditioned media for 14 days, and the osteogenic effect was evaluated by ARS assay. Scale bar = 100 μm. (G) hADSCs were osteogenically induced with different conditioned media for 14 days, and the osteogenic effect was evaluated by ALP assay. Scale bar = 100 μm. (H) qPCR detection of the relative mRNA expression of hADSCs after osteogenic induction with different conditioned media for 14 days, including ALP, BMP2, RUNX2, OPN, OCN, and COLL1. ns: nonsignificant, ∗P < 0.05, ∗∗P < 0.01, ∗∗∗P < 0.001, and ∗∗∗∗P < 0.0001.CM: conditioned media.

T-cells play an important role in immune responses. We further verified the effect of the composite hydrogels on T cells. T cells were co-cultured with the composite hydrogels. After 72 h of culture, the supernatant was collected and ELISA was used to measure the classic cytokines TGF-β, IL-4, IL-17, and IFN-γ secreted by T cell activation (Fig. 10B). The results showed that the expression levels of the control and HPCH@HA groups remained unchanged, but were significantly increased in the HPCH@HA + MgO group. Next, we used PCR to verify the effect of the composite hydrogels on T cells. The results showed that the expression levels of TGF-β, IL-4, IL-17, and IFN-γ genes in the HPCH@HA + MgO group were significantly increased. Next, we extracted the supernatant of T cells from each group as conditioned medium(Fig. 10B). First, we observed the effect of T cell-conditioned medium on HUVECs. Compared with the control group, HPCH@HA + MgO formed obvious blood vessels. At the same time, we added IL-17 and IFN-γ neutralizing antibodies to the HPCH@HA + MgO group. The vascularization effect of the conditioned medium in the HPCH@HA + MgO group was significantly reduced(Fig. 10C). In vivo immunohistochemical staining showed that the proportion of activated T cell surface markers CD3 and CD4 in the HPCH@HA + MgO group increased significantly, whereas there was no significant change in the control and HPCH@HA groups(Fig. 10D and 10E). These results indicate that HPCH@HA + MgO can activate T cells by regulating the bone immune microenvironment and promoting skull defect repair by secreting cytokines that participate in angiogenesis; however, the specific activation mechanism requires further study. At the same time, we observed the effect of macrophages and T cell conditioned culture media on hADSCs osteogenesis. Studies have shown that both conditioned culture media can promote osteogenesis, but macrophage conditioned culture media has better osteogenesis. However, when an appropriate amount of macrophage conditioned culture media is added to the T cell conditioned culture media, the best effect is to promote osteogenesis. This shows that T cells need the help of macrophages to promote osteogenesis(Fig. 10F–H). These results also show that immune cells play an important role in the osteogenesis process through interactions during osteogenesis.

### 3.11 Research on nano MgO loaded complex hydrogels promoting the enrichment of signaling pathways related to macrophage polarization

**Fig. 11:**
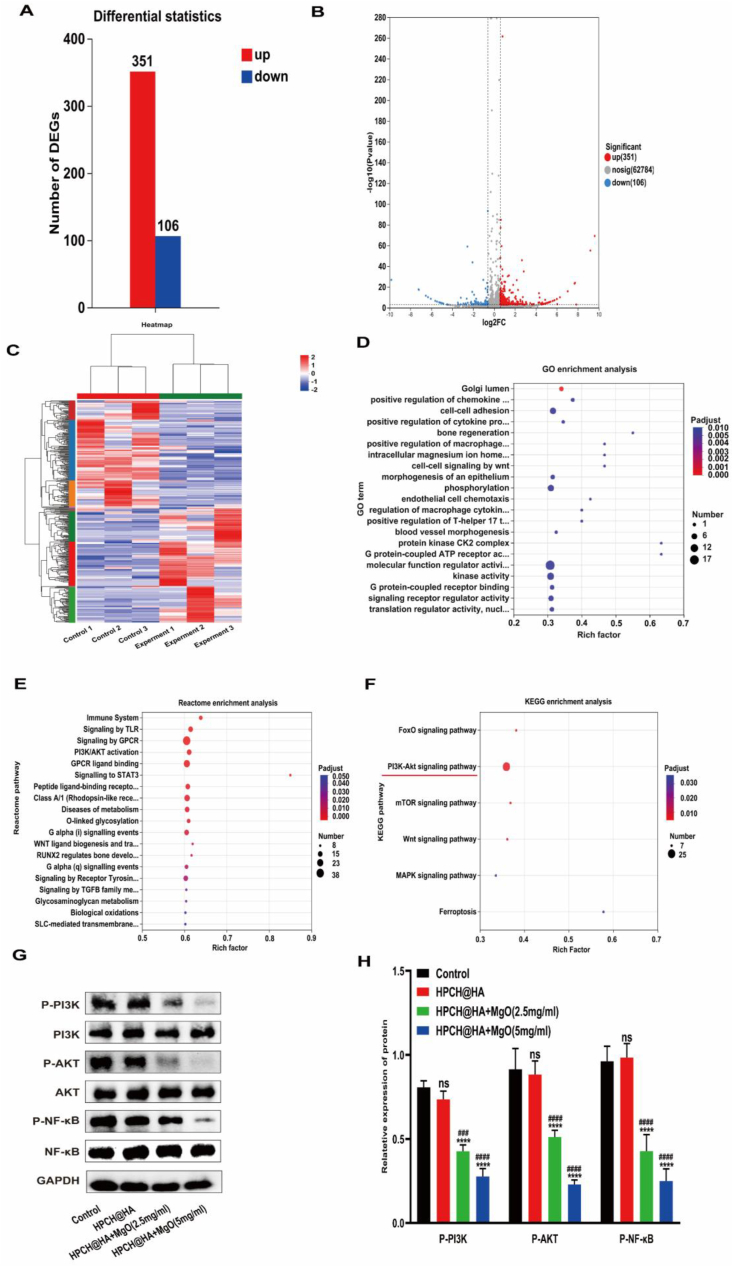
Nano MgO loaded complex hydrogels promotes the enrichment of polarization signaling pathways in macrophages. (A) Statistics of differentially expressed genes between HPCH@HA hydrogel and HPCH@HA + MgO(5 mg/ml) hydrogel. (B) Volcanic maps of differentially expressed genes in the two hydrogel groups. The red dots represent the up-regulated genes in the hydrogel. The blue dots represent the down-regulated genes in the hydrogel. The grey dots represent the unaltered genes between the two hydrogel groups. (C) Heat maps of differentially expressed genes between the two groups of hydrogels. (D) The nano MgO loaded complex hydrogelsgroup upregulated the GO enrichment of the gene. (E) Gene Reactome enrichment of up-regulated genes in the nano MgO loaded complex hydrogels group. (F) The nano MgO loaded complex hydrogels group upregulated the KEGG enrichment of genes. (G, H) Western Blot was used to verify the PI3K-AKT signaling pathway.

To further comprehensively reveal the changes in the expression of genes related to metabolism and macrophage polarization, we conducted RNA transcriptome sequencing. Differential gene analysis showed that 351 genes were upregulated in the HPCH@HA + MgO(5 mg/ml) hydrogel compared with the HPCH@HA hydrogel (Fig. 11A–C). The enrichment of GO genes during biological processes is manifested in the regulation of magnesium ion homeostasis, cell adhesion, chemokine production by macrophages, the regulation of bone regeneration signaling pathways, endothelial cell regeneration, T cell regulatory signaling pathways, and the enrichment of ATP and G protein coupling, which further confirm the immunomodulatory effect of nano MgO loaded complex hydrogel (Fig. 11D). Reactome enrichment shows the enrichment of the immune system, TLR signaling pathway and STAT3 signaling pathway, which are known to be related to immune regulation and macrophage polarization (Fig. 11E). KEGG enrichment suggests the enrichment of known signaling pathways that promote macrophage polarization, such as PI3K-AKT signaling pathway, FOXO signaling pathway, and mTOR signaling pathway (Fig. 11F). We further verified the key proteins of the PI3K-Akt signaling pathway using the Western blot experiment. The results indicated that in the PI3K/AKT pathway, nano MgO loaded complex hydrogel promotes the reprogramming of macrophages from M1 to M2 by inhibiting the phosphorylation of PI3K, AKT and NF-κB (Fig. 11G–H). Overall, the nano MgO loaded complex hydrogels promoted the enrichment of the polarization pathway in macrophages.

### 3.12 Research on the promotion of enrichment of signaling pathways related to osteogenesis by macrophage conditioned medium

**Fig. 12:**
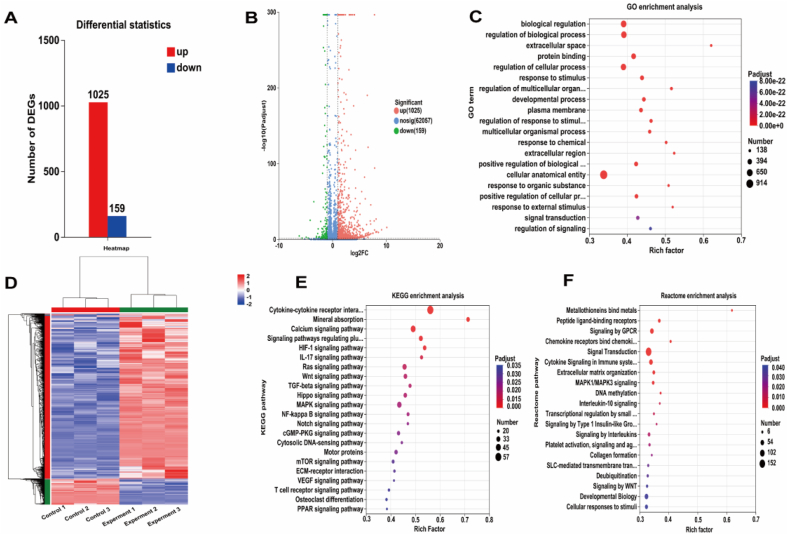
The two groups of macrophage conditioned media promoted the enrichment of osteogenic related signaling pathways. (A) Statistics of differentially expressed genes for osteogenesis induced by conditioned medium in the two groups of macrophages. (B) Volcanic plots of osteogenic differentially expressed genes induced by conditioned medium in the two groups of macrophages. The red dots represent the up-regulated genes in the hydrogel. The blue dots represent the down-regulated genes in the hydrogel. The grey dots represent the unaltered genes between the two hydrogel groups. (C) Heat maps of differentially expressed genes for osteogenesis induced by conditioned medium in the two groups of macrophages. (D) GO enrichment of osteogenic up-regulation genes induced by conditioned medium in macrophages of the nano MgO loaded complex hydrogels group. (E) KEGG enrichment of osteogenic up-regulation genes induced by conditioned medium in macrophages of the nano MgO loaded complex hydrogels group. (F) Gene Reactome enrichment of osteogenic up-regulation genes induced by conditioned medium in macrophages of the nano MgO loaded complex hydrogels group.

To further fully reveal the changes in osteogenic gene expression, we collected the supernatant of macrophages co-cultured with HPCH@HA hydrogel and HPCH@HA + MgO(5 mg/ml) hydrogel and prepared macrophage conditioned medium to induce osteogenic differentiation. We conducted RNA transcriptome sequencing simultaneously. Differential gene analysis showed that 1025 genes were upregulated in HPCH@HA + MgO hydrogel conditioned medium compared with HPCH@HA hydrogel (Fig. 12A–C). Furthermore, the enrichment of GO genes in biological processes is manifested as response to stimulus, developmental process, regulation of biological process, and protein binding (Fig. 12D). (KEGG) enrichment indicates calcium signaling pathway, HIF-1 signaling pathway, WNT signaling pathway, MAPK signaling pathway, and IL-17 Signaling Enrichment of known osteogenesis signaling pathways such as pathways (Fig. 12E). Reactome enrichment shows collagen formation, extracellular matrix organization, and MAPK1/MAPK3 signaling The enrichment of Pathways is known to be related to matrix precipitation and collagen tissue formation during osteogenesis (Fig. 12F). Overall, the macrophage conditioned medium promoted the enrichment of signaling pathways related to osteogenesis.

## 4 Discussion

The host inflammatory response induced by the implanted biomaterials plays an important role in tissue regeneration, and immune cells play an important role in this process [26]. In the early stages of tissue engineering, the development of bone defect repair materials usually involves activating stem cells for osteogenesis through different methods, while ignoring the interaction between biomaterials and the host immune system. The early immune response of immune cells near biomaterials determines the fate of the implants in the body [27]. Biomaterials with certain chemical structures or mechanical properties can regulate immune responses related to bone metabolism through immune cells such as macrophages, thereby maintaining the bone metabolism balance near implanted materials through bone immune regulation [28]. However, recent studies have found that immune cells such as macrophages, T lymphocytes, B lymphocytes, and neutrophils play an important role in regulating the balance of bone metabolism by regulating the generation, maturation, and differentiation of stem cells; promoting angiogenesis through cytokines; and regulating inflammatory responses during bone metabolism [[29], [30], [31], [32]]. Therefore, the interaction between bone cells and immune cells, development of new biomaterials with bone immune regulation function, and targeted regulation of the interaction between immune cells and stem cells to promote osteogenesis and angiogenesis are hot topics in the field of orthopedics and materials and have important clinical significance in solving the difficult problems faced in clinical practice, such as large bone defects and tendon-bone healing.

This study aimed to explore the osteoimmune effects of the HPCH@HA + MgO hydrogel system on in situ bone regeneration and angiogenesis. The designed hydrogel system has high hydrophilicity and a porous three-dimensional structure; therefore, it can slowly release Mg^2+^ in the local tissue microenvironment. At the same time, this hydrogel has excellent bone affinity, can rapidly solidify and cover bone defects in the body, and can be used to slowly release Mg^2+^ to promote bone healing (Fig. 1E). Electron microscopy revealed that the new hydrogel could effectively load Mg^2+^ and that the pores were uniform (Fig. 1A–D). Studies have confirmed that Mg^2+^ can stimulate M2 polarization of macrophages [33]. In vitro and in vivo experimental data showed that the HPCH@HA + MgO hydrogel system produced good anti-inflammatory properties, an osteoimmune environment, regulated macrophage polarization, and activated regulatory T cells, thereby promoting stem cell osteogenic differentiation and HUVECs angiogenesis, and multiple effects promoted bone defect repair.

First, to simulate the inflammatory response of Mg^2+^ to macrophages in the human immune microenvironment, we first used THP-1 cells inoculated onto the hydrogel surface for in vitro experiments to detect macrophage polarization indicators, cytokine secretion, and gene expression. Among the many immune cells in the body, macrophages are one of the most important for regulating innate immune responses and tissue remodeling. These cells recruit other immune cells to the site of injury by secreting inflammatory factors. In addition, the response of macrophages to the surrounding microenvironment prompts them to transform into the M1 and M2 phenotypes, which is beneficial for tissue regeneration [[34], [35], [36], [37]]. As shown by the immunofluorescence results (Fig. 4), the HPCH@HA + MgO hydrogel upregulated the expression of the M2 surface marker CD206 and downregulated the expression of the M1 surface marker CD86, indicating that the HPCH@HA + MgO hydrogel is conducive to the transformation of the M2 phenotype. Flow cytometry showed that the proportion of M2 macrophages in the nano MgO loaded complex hydrogel group was significantly increased, that of the M1 type was significantly reduced, and that of the M2 phenotype in the nano MgO loaded complex hydrogels 5 mg/ml group was significantly higher compared with that in the 2.5 mg/ml group. The transcriptomic results also indicated that the nano MgO loaded complex hydrogel could promote the enrichment of polarization-related pathways in macrophages, such as the PI3K-AKT signaling pathway and the TLR signaling pathway (Fig. 11).Studies have shown that magnesium ion concentration has a significant effect on macrophage activity [[38], [39], [40], [41], [42]]. However, determination of the concentration of Mg^2+^ in different macrophage phenotypes requires further investigation.

Next, we used ELISA to detect cytokine secretion by macrophages to further verify the ability of the HPCH@HA + MgO hydrogel to regulate the immune microenvironment. Macrophages in the HPCH@HA + MgO group secreted higher concentrations of anti-inflammatory factors (Arg-1, VEGF, TGF-β) and reduced the expression of pro-inflammatory cytokines TNF-α, IL-6, and IL-1β (Fig. 4). Studies have shown that TNF-α, IL-6, and IL-1β promote osteoclastogenesis and inhibit osteogenic differentiation [[43], [44], [45]]. The classical cytokines Arg-1, VEGF, and TGF-β secreted by M2 cells can promote the osteoblast differentiation of stem cells and the angiogenesis of endothelial cells. Specifically, TGF-β plays an important role in osteogenic differentiation. TGF-β can recruit and attract stem cells and promote osteogenesis through the SMAD signaling pathway. New blood vessels are essential for the formation of new bone [46]. Previous studies have shown that VEGF, FLT, and KDR promote angiogenesis and vascularization during tissue regeneration. In addition, VEGF, an angiogenic growth factor, not only enhances the migration of endothelial cells but also binds to VEGF receptors on the surface of endothelial cells to promote angiogenesis, further promoting osteogenesis [[47], [48], [49]]. In this study, the conditioned medium of M2 macrophages activated by HPCH@HA + MgO hydrogels enhanced the vascularization of endothelial cells in vitro (Fig. 5), which may be due to the increased expression of the aforementioned pro-regenerative cytokines.

Studies have shown that during bone injury, macrophages are rapidly recruited to damaged areas, releasing active factors that have a significant chemotactic effect on stem cells, thereby promoting stem cell colonization and differentiation [50]. Therefore, we evaluated the osteogenic ability of hADSCs cultured in a conditioned-bone microenvironment (Fig. 6). The differentiation and mineralization abilities of hADSCs in the HPCH@HA + MgO macrophage-conditioned medium were significantly enhanced, and the expression of osteogenic genes was significantly increased. Simultaneously, the migration ability of stem cells in the HPCH@HA + MgO group was significantly increased, which may be related to the regulation of the bone tissue immune microenvironment by magnesium. The possible reason is that TGF-β activates the expression of osteogenic-related genes ALP, BMP2, RUNX2, OPN, OCN, and COLL1 [51,52]. The transcriptomic results also indicated that the macrophage conditioned medium could promote the enrichment of osteogenesis-related pathways such as the WNT signaling pathway, calcium ion signaling pathway,HIF-1 signaling pathway, and MAPK signaling pathway (Fig. 12).In addition, studies have shown that Mg^2+^ can promote osteogenesis and angiogenesis in stem cells. however, the local Mg^2+^ concentration must be controlled. High concentrations can lead to bone loss [38,53]. A possible reason for this is that Mg^2+^ acts through TRPM7 on the cell surface [54,55]. However, when magnesium-based implants are used in animal models, due to their rapid degradation in the physiological environment, a large amount of Mg^2+^ is released, leading to osteolysis around the implant. Moreover, excessive Mg^2+^ has adverse effects on bone metabolism in the body, which may result in impaired bone mineralization and bone diseases [56]. Therefore, attention should be paid to the concentration of Mg^2+^ in practical applications. Studies have shown that Mg^2+^ in the concentration range of 6–1000 ppm can promote the proliferation and differentiation of osteoblasts and stem cells, with no damage to osteoblast-related cells. Using pure magnesium implant extract, 10 mM Mg^2+^ is the critical concentration that does not affect cell proliferation, and 15 mM Mg^2+^ can be considered the overall safe dose. When magnesium salts such as MgCl_2_ or MgSO_4_, were used as Mg^2+^ sources, the tolerated dose of Mg^2+^ was relatively high, and 20 mM Mg^2+^ did not negatively affect cell viability. The concentration of Mg^2+^ released in our study ranged from 50 to 200 ppm, which is within a safe and reasonable concentration range [[57], [58], [59]].

T-cells play an important role in adaptive immune responses. Studies have found that T cells promote bone defect repair by secreting cytokines such as TGF-β, IL-17, and IFN-γ, and also play an important role in the formation of the inflammatory microenvironment. However, there are few reports on the regulation of the biological behavior of T cells by Mg. Our ELISA and PCR results show that loading Mg^2+^ in the HPCH@HA hydrogel system can effectively promote T cell activation to secrete TGF-β, IL-4, IL-17, and IFN-γ, and then promote angiogenesis [[60], [61], [62], [63]]. We found that HPCH@HA-MgO has a better ability to promote T cell secretion of IL-17 and IFN-γ than does HPCH@HA. The supernatant of T lymphocytes co-cultured with HPCH-HA-MgO also showed better effects in promoting angiogenesis. After adding IL-17 and IFN-γ neutralizing antibodies to the nano MgO loaded complex hydrogels group of T cell conditioned media (Fig. 10), tube formation abilities were significantly reduced, indicating that Mg^2+^ can activate T cells. At the same time, magnesium-ion hydrogels induced a higher proportion of CD3 and CD4 positive T cells at the injury site, effectively promoting skull repair [64]. Our results indicate that M2 macrophages can enhance the osteogenic effect of T cells (Fig. 10). Meanwhile, researchers have constructed sugar peptide hydrogel that can recruit and mediate the polarization of M2 macrophages, and further activate the CD3^+^CD4^+^ T cell immune response that promotes regeneration, and enhance the cell-material interface interaction, and promote angiogenesis in the local tissue environment, thereby facilitating wound repair and regeneration [65]. The double-layer alginate hydrogel can guide the polarization of macrophages to the immunosuppressive phenotype M2, thereby activating Th2 helper T cells to promote wound healing [66]. Existing studies have shown that macrophages can all promote the production of IL-17 by CD4^+^ T cells [67,68], which affects bone formation. Additionally, it has been found that in the case of CD4^+^ CD25^+^ regulatory T cells, macrophages can survive and exhibit an anti-inflammatory phenotype [69], all of which are consistent with our research results. However, some studies have shown that Treg-Exo can promote the polarization of macrophages to the M2 type, thereby promoting the proliferation of bone BMSCs and bone formation, and promoting fracture healing [70], which may be due to the different functional states of the two cells at different healing stages. Therefore, the interaction between macrophages and T cells in different internal environments is worthy of in depth exploration in the future.

Studies have shown that macrophages, especially type M2, are regarded as core regulators in angiogenesis. They promote angiogenesis by generating angiogenic growth factor families such as VEGF, EGF, and FGF, angiogenic chemokines CXCL8 and CXCL12, and angiogenesisn related factors TGF-β, HIF-1α, and thymidine phosphorylase. These secretory factors also enhance the migration and proliferation of endothelial cells [[71], [72], [73]]. The role of T lymphocytes is more indirect and dependent on subsets, and their functions have vascular-promoting or anti-vascular characteristics. TH1 cells can polarize M2-like TAM into M1 macrophages and induce the maturation of DC in the TME, thereby inhibiting tumor angiogenesis [74]. The secretion of IL-4, IL-13 by Th2 cells can induce polarization of M2-type macrophages and indirectly promote angiogenesis. Regulatory T cell(Treg) supports endothelial cell recruitment and dilation of angiogenesis by secreting VEGF, IL-4 and IL-5, and can also indirectly promote angiogenesis by activating M2 macrophages [75]. Cytotoxic T cells may inhibit angiogenesis by killing endothelial cells or secreting angiostatin [76]. Studies have found that γδT cell deficiency in mice leads to an increase in the accumulation of white blood cells and M1-like macrophages, resulting in a decrease in the number of endothelial cells and impaired angiogenesis [77], and PD-002 promotes angiogenesis through T cell dependent M2 macrophages [78]. Therefore, in the process of promoting angiogenesis, both macrophages and T lymphocytes play important roles, but their mechanisms of action and dominance vary depending on the physiological or pathological background. In most cases, macrophages, especially the M2 type, are the "executors", playing a more direct and core role. T cells as "regulators", indirectly affect angiogenesis by regulating the immune microenvironment and intercellular information transmission.

We verified the ability of the nano MgO loaded complex hydrogels to regulate the local tissue immune microenvironment in vivo. Therefore, we constructed a skull defect model to evaluate the bone-repair effect. When the magnesium hydrogel was implanted, macrophages were recruited to the surface of the biomaterial. Within 48 h of bone injury, a large number of macrophages, mainly pro-inflammatory M1 type, will be rapidly recruited to participate in the clearance of necrotic tissue and the initiation of inflammatory responses. The period within 72 h is the transitional period of the inflammatory peak. During subsequent repair, the M1/M2 ratio will gradually shift towards M2 dominance. Studies have shown that electrospinning naringin microbeads can promote the increase of M2 polarized CD206+ cells within 3 days. Promote the repair of tissue bone defects [79], which is consistent with our research (Fig. 8). At 8 weeks,the immunofluorescence and immunohistochemistry results further confirmed that the magnesium hydrogel could induce a higher proportion of CD206-positive cells (M2 macrophages) and a lower proportion of CD86-positive cells (M1 macrophages) at the injury site (Fig. 9). An increase in the M2/M1 ratio indicated that a good anti-inflammatory and immune microenvironment was achieved. The transition of macrophages from the pro-inflammatory M1 phenotype to the anti-inflammatory M2 phenotype is accompanied by the secretion of anti-inflammatory cytokines, which induces a good immune microenvironment that is conducive to bone immune regulation. In contrast, the two control groups showed less new bone formation 8 weeks after surgery, which was due to the high proportion of M1 phenotype leading to the production of pro-inflammatory cytokines TNF-α, IL-6, and IL-1β in the bone tissue microenvironment; the continued presence of the M1 phenotype can lead to chronic inflammation of the local tissue, resulting in poor bone healing [80].

In the three-dimensional reconstruction model displayed by microCT, the bone defect in the nano MgO loaded complex hydrogels group healed 8 weeks after surgery. The BMD, BV/TV, and bone volume results showed obvious new bone mineralization and improved mechanical properties. In addition, the results of HE, Goldner, and Masson staining showed that the nano MgO loaded complex hydrogels induced a large amount of bone formation in the early stages and accelerated the bone healing process (Fig. 9). This also indicates that the ability of pure hydrogel to induce bone repair is limited, while the addition of nano MgO can significantly improve the osteogenic effect. This might be because magnesium ions promote bone regeneration through multiple pathways such as the WNT pathway and the MAPK/ERK pathway, promoting its proliferation, migration and adhesion, osteogenic differentiation, and thereby promoting bone healing [81,82]. Immunohistochemistry also showed that the expression of BMP2 and OPN in the nano MgO loaded complex hydrogels group increased significantly, indicating stronger bone formation ability [83,84]. In addition, immunohistochemistry showed that the vascular indicators VEGF and FLT in the magnesium-ion hydrogel group increased significantly. Studies have shown that the skeletal vascular system plays an important role in the development, repair, and regeneration of bones. This dysfunction can lead to bone aging and diseases [85,86]. At the same time, the magnesium-ion hydrogel that we constructed can solidify rapidly in vivo to provide mechanical support and can be used alone for bone defects of a critical size.

In recent years, the research on bone biomaterials has been making continuous progress. For instance, it has been found that calcium phosphate nanocerceramic (CaP) and its biomimetic precipitated nanocrystal calcium phosphate (BpNcCaP) have a similar composition to natural bone tissue, possess good degradability, no cytotoxicity, and appropriate hardness, and can induce osteogenesis by regulating the polarization of macrophages. These characteristics make it show great potential in bone defect repair [87,88]. Meanwhile, given the complex microenvironment in the bone repair stage, some researchers have constructed multifunctional hydrogel systems to meet the spatiotemporal management requirements of the immune microenvironment, revascularization and osteogenic differentiation. The hydrogel composed of black phosphorus nanosheets(BPPD) loaded with deferriamine (DFO) and methacrylate gelatin/methacrylate sodium alginate (GA/BPPD) has excellent near-infrared (NIR)/PH dual response characteristics. During bone regeneration, it can eliminate excessive ROS and alleviate local inflammatory responses, ensuring the rapid transformation of macrophages from M1 to M2 [89]. Subsequently, the sequential release of the angiogenic factor DFO and the osteogenic factor PO_4_^3−^ is achieved. The NIR trigger has both photothermal therapeutic effects, ultimately realizing the synergistic therapeutic effect of immune regulation, angiogenesis and osteogenesis. Another hydrogel system (HG/MPa) has NIR light response and spatiotemporal specificity. It is composed of Ti3C2Tx MXene (MP) nanosheets coated with polydopamine, aFGF and hydroxypropyl chitosan/gelatin (HG), and has good mechanical and biological properties. Under the action of NIR, the initial complete release of aFGF can be achieved, rapid angiogenesis can be induced, and cell adhesion, proliferation and migration can be promoted [90]. Meanwhile, the excellent photothermal effect of MP can synergistically promote bone formation. Meanwhile, in view of the disordered pathological microenvironment in diabetic bone defects, researchers constructed a multifunctional hydrogel. This system integrates copper-modified MXene (MC) nanosheets, methacrylic gelatin and alginate dopamine (GAD), and has good physical and chemical properties. The thermotherapeutic effect of MC nanosheets enhances bone repair, and the degradation products, titanium-based substances, can promote the growth of osteoblasts and endothelial cells. Meanwhile, hydrogels can regulate the sustained release of Cu^2+^ at the injury site and effectively eliminate excessive ROS, thereby inhibiting the inflammatory response. These characteristics are conducive to reshaping the disordered immune microenvironment and accelerating the bone healing process [91]. These studies all indicate that the intelligent multifunctional hydrogel system has a bright application prospect in the treatment of bone defects.

In terms of novelty, the magnesium-ion composite hydrogel we constructed is soft and elastic, capable of better adhering to complex tissue surfaces. It's thermosensitive properties can rapidly solidify in the body, providing mechanical support and creating an anti-inflammatory, osteogenic and vascularizing microenvironment locally. The high water content can provide a moist microenvironment for cells, which is conducive to cell growth and promoting tissue repair. The preparation process is relatively simple and can be achieved at room temperature through methods such as solution mixing and cross-linking [92]. The hydroxyapatite coating is relatively brittle in texture, prone to cracking and even peeling, and lacks moisture retention ability. This may cause dehydration of the tissue cells in contact with it, affecting the function of the cells and the healing of the tissues. Preparation often requires rather complex techniques and equipment, such as physical vapor deposition methods [93,94]. Meanwhile, the stacking mode between magnesium-containing PLGA microspheres makes the space for cell growth relatively limited. Moreover, in practical applications, there may be situations where the release is too rapid in the early stage or insufficient in the later stage, and it is mainly due to the sustained-release effect of magnesium ions, relatively lacking this multi-component and multi-dimensional biological activity synergistic effect [95]. So nano MgO loaded HPCH@HA hydrogels have great potential in bone defect repair.

Of course, the mouse borderline skull defect model also has some drawbacks. For example, skull defects can be used as a model of intramembranous bone formation, which may not be suitable for biomaterials or strategies for endochondral bone formation. It is difficult to evaluate the mechanical properties of regenerated bone in a skull defect because skull defects are small and the healing site does not bear weight. Finally, current cranial regeneration strategies often fail to generate new, highly vascularized bone [96,97]. However, considering the goals of biomaterials or bone regeneration strategies, rapid process of bone repair, and relatively simple surgical procedure in mice, the mouse skull defect model can be used as a rapid and high-quality method for in vivo evaluation of bone regeneration. Our study also demonstrates that the nano MgO loaded complex hydrogels has excellent integration with the bone host environment and is a good immune-responsive biomaterial to guide successful osteogenesis and angiogenesis, which has great potential for clinical applications. In addition, it can be used to repair skull defects without the use of auxiliary exogenous growth factors or cells, thereby improving its clinical transformability.

## 5 Conclusion

Our results showed that when the HPCH-HA-MgO hydrogel was used to treat bone defects, bone immune regulation was essential. The composite hydrogel can simulate the immune microenvironment of bone tissue, regulate Mg^2+^ release, and concurrently regulate the transformation of macrophages to the M2 phenotype and activate T lymphocytes, thereby secreting pro-regenerative cytokines, recruiting repair cells (like hADSCs and HUVECs), promoting the angiogenesis of endothelial cells, and promoting bone healing. Mg^2+^ release can induce macrophage M2 phenotype conversion, accompanied by high levels of Arg-1 secretion, enhanced TGF-β expression to promote osteogenesis, and increased VEGF to promote angiogenesis. In addition, cytokines such as TNF-α, IL-6, and IL-1β were also significantly inhibited. At the same time, activated T cells release TGF-β, IL-4, IL-17, and IFN-γ to promote osteogenesis and angiogenesis. This favorable anti-inflammatory and osteogenic tissue microenvironment can promote the differentiation of hADSCs by upregulating ALP, OCN, OPN, COLL1, RUNX2, and BMP2, and promote angiogenesis by upregulating VEGF and FLT, thereby accelerating subsequent in vivo bone regeneration. Given the increasing recognition of the importance of regulating immune-inflammatory responses in tissue regeneration, we believe that a better understanding of the interactions between biomaterials and the immune system will provide more clinically valuable solutions for large bone defects.

## CRediT authorship contribution statement

**Feifei Ni:** Writing – original draft. **Longkang Chang:** Data curation. **Yizhong Peng:** Formal analysis. **Dongxu Li:** Methodology, Data curation. **Dong Wu:** Investigation. **Kanglu Li:** Software. **Xin Zhang:** Software. **Xulin Jiang:** Supervision. **Zengwu Shao:** Supervision. **Yangyang Chen:** Supervision. **Hong Wang:** Supervision.

## Availability of data and materials

The data that support the fndings of this study are available from the corresponding author upon reasonable request.

## Ethics approval and consent to participate

All human samples and animal experiments were obtained with the informed consent of the patients and the protocols were reviewed and approved by the Ethics Committee for the Use of Human Subjects of the Union Hospital Affiliated to Huazhong University of Science and Technology (Issue No: UHCT-IEC-SOP-016-03-01. IACUC Number: 4008), and the animal experiments in accordance with the National Research Council's Guide for Care and Use of LaboratoryAnimals.

## Funding

This work was supported by the 10.13039/501100001809National Natural Science Foundation of China (81672166).

## Declaration of competing interests

There are no conflicts to declare.

## Abbreviations

HPCH:hydroxypropyl chitin hydrogel; hADSC:human adipose-derived stem cell.

HUVEC:human umbilical vein endothelial cell; IFN-γ:interferon-gamma.

IL-17:interleukin-17; TGF-β:transforming growth factor-β; HA:Hyaluronic acid.

ICP–OES:inductively coupled plasma optical emission spectroscopy.
